# Supplementary material for: Orbital-resolved imaging of coherent femtosecond exciton dynamics in coupled molecules
Source: Nat Commun. 2026 May 15;17:6455. doi: 10.1038/s41467-026-73191-0 (PMC13376558; doi:10.1038/s41467-026-73191-0)
Supplement: Supplementary file 1 — Supplementary Information [file 41467_2026_73191_MOESM1_ESM.pdf]

Supplementary Information for

Orbital-resolved imaging of coherent femtosecond exciton dynamics in coupled molecules

Yang Luo[1,2]†, Shaoxiang Sheng[1,3]†, Michele Pisarra[4,5]†, Caiyun Chen[1], Fernando Martín[6,7]*, Klaus Kern[1,8], Manish Garg[1,]*

[1]Max Planck Institute for Solid State Research, Heisenbergstr. 1, 70569 Stuttgart, Germany

[2]Hefei National Laboratory, University of Science and Technology of China, Hefei, 230088, China

[3]Tsientang Institute for Advanced Study, Hangzhou, 310024, China

[4]Dipartimento di Fisica, Università della Calabria, Via P. Bucci, cubo 30C, 87036, Rende (CS), Italy

[5]INFN-LNF, Gruppo Collegato di Cosenza, Via P. Bucci, cubo 31C, 87036, Rende (CS), Italy

[6]Instituto Madrileño de Estudios Avanzados en Nanociencia (IMDEA Nano), Faraday 9, Cantoblanco, 28049 Madrid, Spain

[7]Departamento de Química, Módulo 13, Universidad Autónoma de Madrid, 28049 Madrid, Spain

[8]Institut de Physique, Ecole Polytechnique Fédérale de Lausanne, 1015 Lausanne, Switzerland

*Corresponding authors. Email: mgarg@fkf.mpg.de and fernando.martin@imdea.org

†These authors contributed equally to this work: Y. Luo, S. Sheng and M. Pisarra

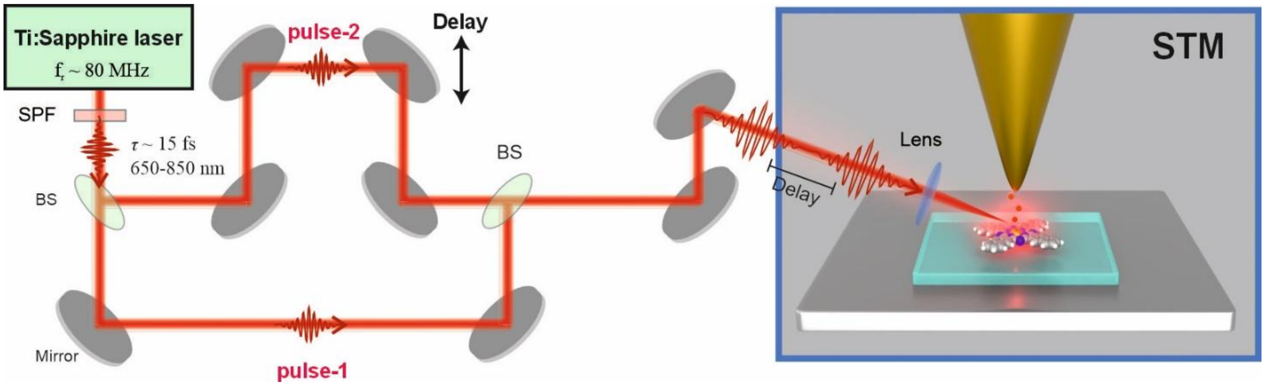

Supplementary Figure 1 | Experimental setup. In the time-resolved measurements, two time-delayed laser pulses (pulse-1 and pulse-2) in the spectral range spanning from ~ 650 nm to 850 nm were generated by traversing the ultra-broadband laser pulses from a Ti:Sa oscillator through an ~ 850 nm shortpass filter. The laser pulses were combined and focused onto the STM junction by a biconvex lens of focal length of 7.5 cm. BS: Beam splitter; SPF: Shortpass filter.

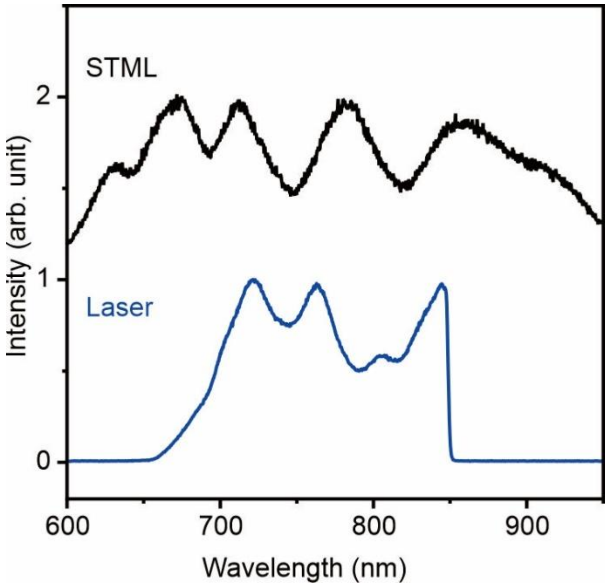

Supplementary Figure 2 | Comparison of the STM induced electroluminescence (STML) spectrum of the plasmonic junction (black curve) with the spectrum of the incident laser pulses (blue curve). STML spectrum was measured on the bare Ag(111) surface at a bias of 2.5 V and tunneling current of 100 pA.

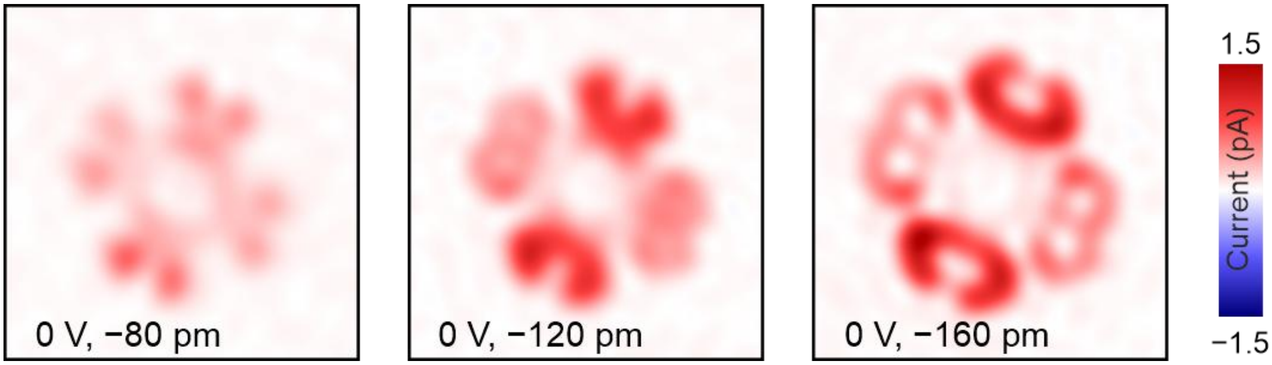

Supplementary Figure 3 | Ultrafast photocurrent images (4.0 nm × 4.0 nm) of a single CuNc molecule on a 4ML thick NaCl film acquired with the open feedback loop (constant-height mode) at various heights of the nanotip as annotated in each individual panel. A laser power of 100 μW was used in the measurements. Before acquiring the images, the nanotip was stabilized at 1 V and 2 pA on the molecular lobe (red cross in Fig. 1c, main-text), and then approached by 80 pm (left-most panel), 120 pm (middle panel) and 160 pm (right-most panel) at 0 V. At closer tip-molecule distances (-160 pm, right-most panel), the photocurrent image is visibly different from the imaging at larger tip-molecule distance (-80 pm, left-most panel). This observation could stem from the stronger tip-molecule hybridization at closer distances (Ref. 30, main-text), moreover, the contributions of different photocurrent channels (Fig. 2c, main-text) will also evolve with tip-molecule distance.

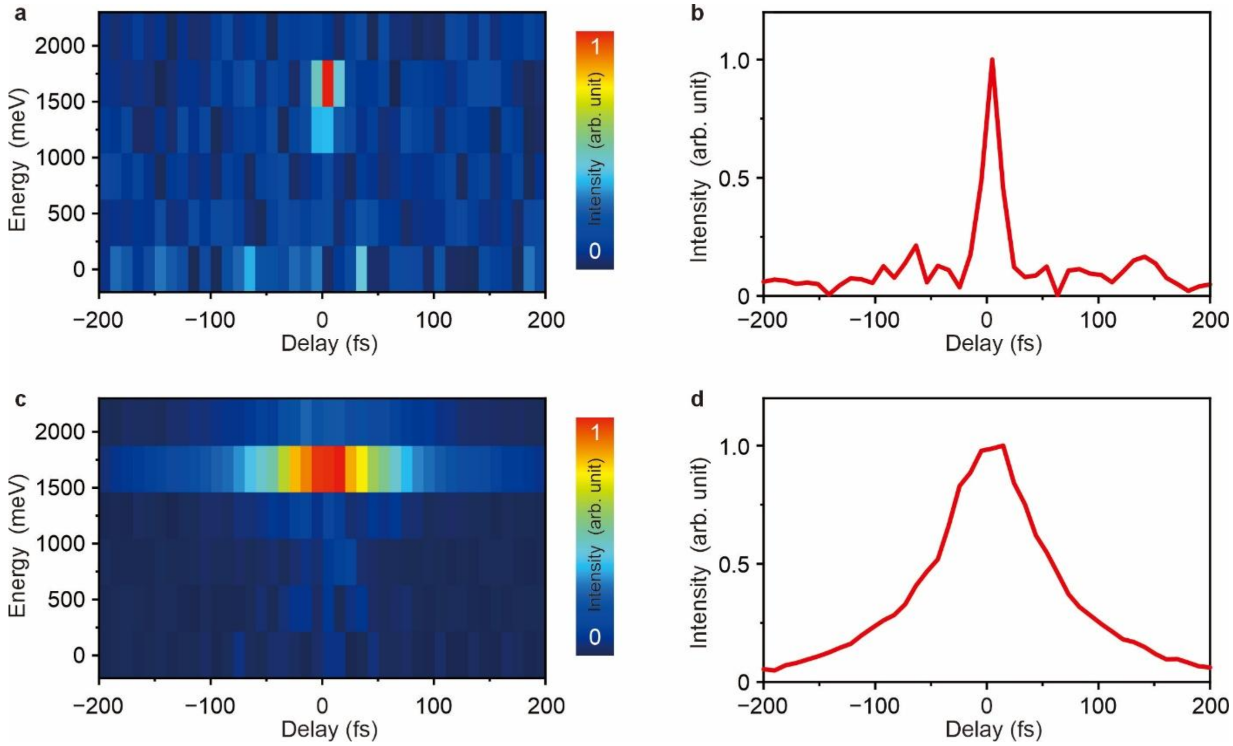

Supplementary Figure 4 | Time-frequency analysis of the time-resolved photocurrent signal measured on bare NaCl (**a**) and on a CuNc (**c**) molecule, respectively**.** The analysis is performed on the time-domain signal shown in Fig. 3d (NaCl) and Fig. 3b (CuNc) of the main-text. **a, b,** Short-time Fourier transform spectrum was obtained using a 15-point (20 fs) Kaiser window with a shape parameter of 4. **b, d,** Temporal evolution of the intensity of the spectral envelope at ~ 1700 meV. In case of bare NaCl (Fig. 3d, main-text), time-frequency analysis reveals a broad spectral distribution (a), which is localized in the temporal window of ~ 15 fs (b). However, in the case of time-resolved photocurrent measurements on the CuNc molecule, a single frequency component corresponding to the excitonic peak can be seen in the broad temporal window extending up to ~200 fs (c, d). No frequency component corresponding to the carrier frequency of the incident laser pulses can be seen in the time-frequency plot, attesting to the absence of the thermal effects in the experiments. Presence of frequency components remnant of the carrier frequency of the laser would have indicated thermal artefacts. At the pulse energies used in the current experiment, no photodesorption or tip-induced damage of the molecule was observed.

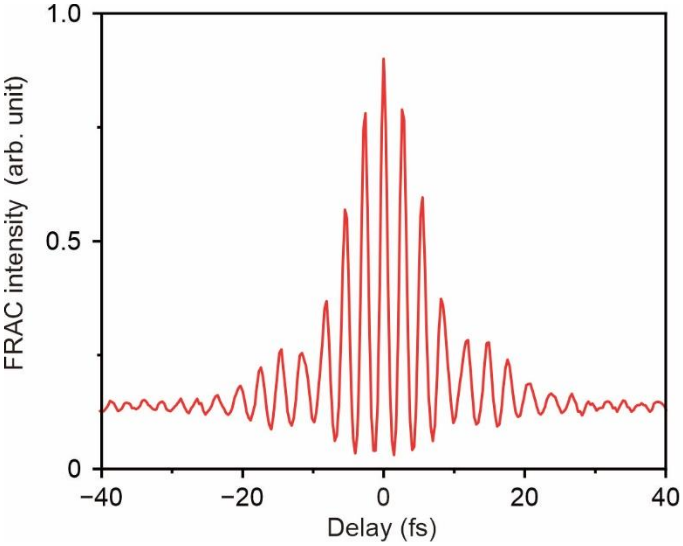

Supplementary Figure 5 | Fringe-resolved autocorrelation trace of laser pulses (τ_{FWHM} ~ 10 fs) incident on the STM junction. A second harmonic generation based fringe-resolved autocorrelator (FRAC) with an ~ 20 μm thick BBO crystal was used to measure the duration of the pump and probe pulses with identical dispersion as in the optical path to the STM junction.

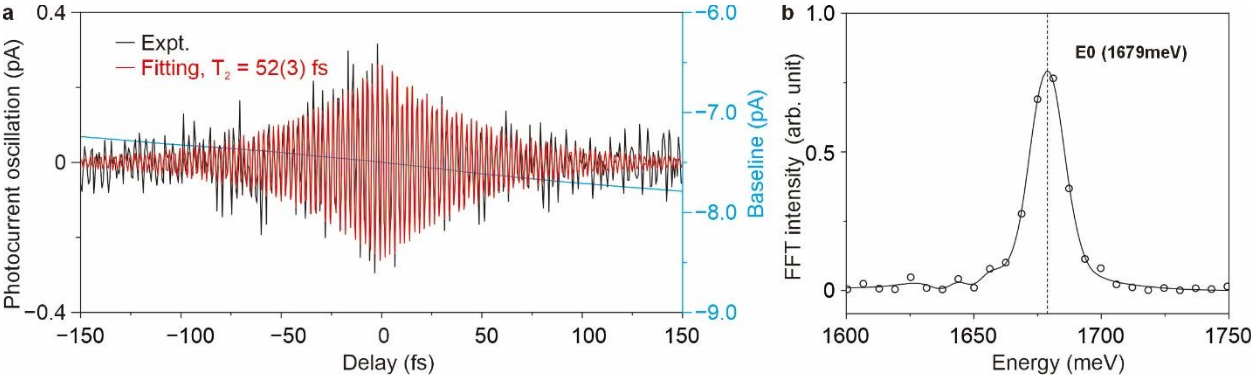

Supplementary Figure 6 | Quantum decoherence of excitons in a single CuNc molecule on a 2 ML NaCl on the Ag(111). a, Ultrafast photon-induced tunneling current measured on the lobe of a single CuNc molecule present on top of a 2ML thick NaCl island as a function of the delay between pulse-1 and pulse-2. The oscillatory component (black curve) of the photocurrent was extracted by subtracting a smoothed baseline (blue curve) from the raw time trace. The nanotip was stabilized at 1 V and 2 pA on the molecular lobe and was further approached by 260 pm before starting the time-resolved measurements. The decoherence time T_2 was determined by fitting the time trace to the function, $I = \cos(\omega_0\tau)*\exp(-|\tau|/T_2)$, where ω_0 corresponds to the excitonic absorption peak, and τ is the time delay between the pulses. **b,** Fast Fourier transformation (FFT) spectrum of the time trace in **a**, indicating the position of the excitonic peak in the monomer.

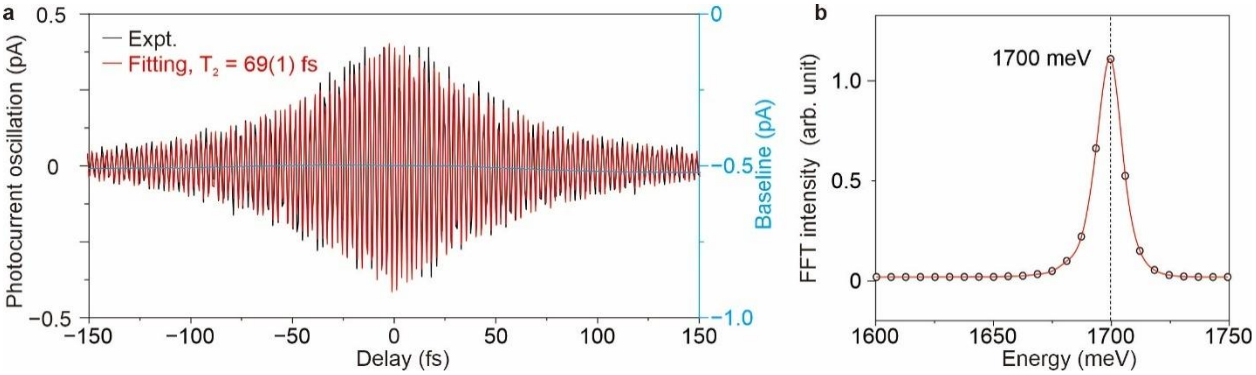

Supplementary Figure 7 | Quantum decoherence of excitons in a single CuNc molecule on a 4ML thick NaCl film on Ag(111). a, Time-resolved photocurrent measured at the lobe position of a single CuNc molecule present on top of a 4ML thick NaCl film. The nanotip was stabilized at 1 V and 2 pA on the molecular lobe, then the bias was changed to −1 V and the nanotip was further approached by 80 pm before starting the time-resolved measurements. The oscillatory component (black curve) of the photocurrent was extracted by subtracting a smoothed baseline (blue curve) from the raw time trace. **b,** Fast Fourier transformation (FFT) spectrum of the time trace in **a**, indicating the position of the excitonic peak in the monomer. The excitonic peak in the case of a molecule on 4 ML is slightly blue shifted to ~ 1700 meV compared to the peak position in 2 ML (~ 1679 meV) and 3 ML thick NaCl (~ 1687 meV), likely due to lower electromagnetic screening of the molecule from the underlying Ag(111) substrate.

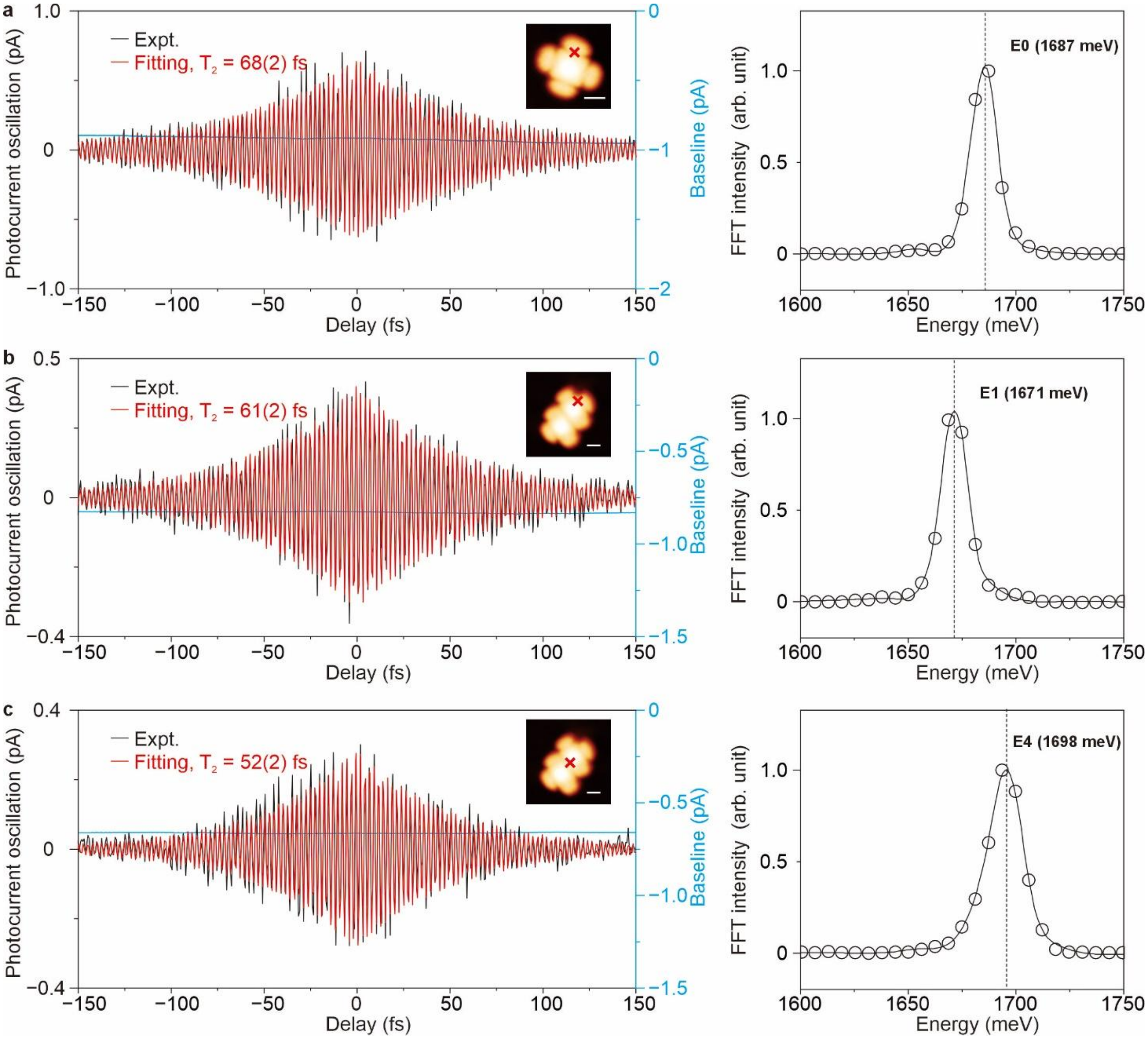

Supplementary Figure 8 | Retrieving decoherence time of excitons in monomer and dimer of CuNc molecules from the time-resolved measurements. a, **b**, **c**, Left Panels: Exciton decoherence times (T2) determined by fitting the time-resolved photocurrent traces obtained with the nanotip of the STM positioned on the lobe of the monomer (**a**), one edge of the dimer (**b**), and on the center of the dimer (**c**). The oscillatory component (black curve) of the photocurrent time traces were extracted by subtracting a smoothed baseline (blue curve) from the raw time traces. The decoherence time T_2 was determined by fitting the photocurrent time trace to the function, $I = \cos(\omega_0\tau)*\exp(-|\tau|/T_2)$, where ω_0 corresponds to the excitonic absorption peak, and τ is the time delay between the pulses (pulse-1 and pulse-2). The decoherence time of a monomer is estimated to be ~ 70 fs. The lowest energy peak of molecular dimer, i.e. E1, exhibits a shorter decoherence time of ~ 61 fs, whereas the decoherence time of the highest energy peak, i.e. E4, is even shorter (~ 52 fs). The right panels in **a**, **b** and **c** display the fast Fourier transform (FFT) spectra of the corresponding time traces.

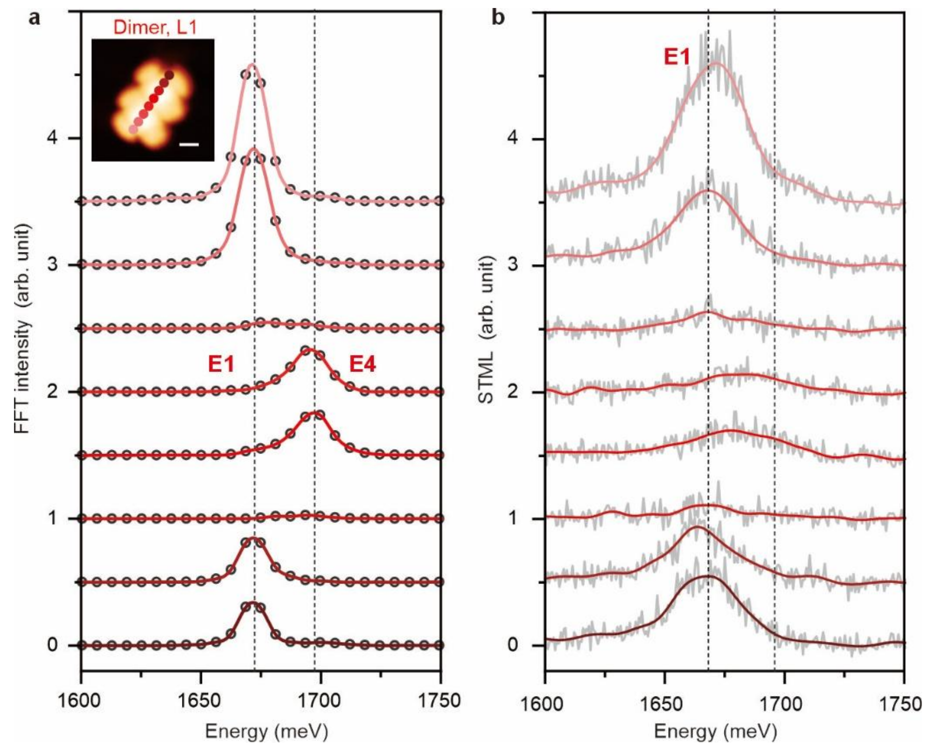

Supplementary Figure 9 | Comparison of spectra obtained from FFT of the time-resolved traces and STML. a, A series of FFT spectra retrieved from time-resolved photocurrent measurements at eight equidistant positions of the nanotip over the CuNc dimer (red dots in the inset of **a**), adopted from Fig. 5a of the main-text. **b,** STML spectra measured at the identical positions of the nanotip over the dimer as in **a** at a bias of −2.2 V and tunneling current of 50 pA. An integration time of 30 seconds was used for all the spectra. The spectra in the STML measurements only show the bright excitonic state (E1, 1671 meV), whereas the dark excitonic state (E4, 1698 meV) was not observed at the center of the molecular dimer. This is likely due to very short decoherence time of the dark states, making them hard to detect in emission based spectroscopy. The spectra have been vertically shifted in **a** and **b** for clarity.

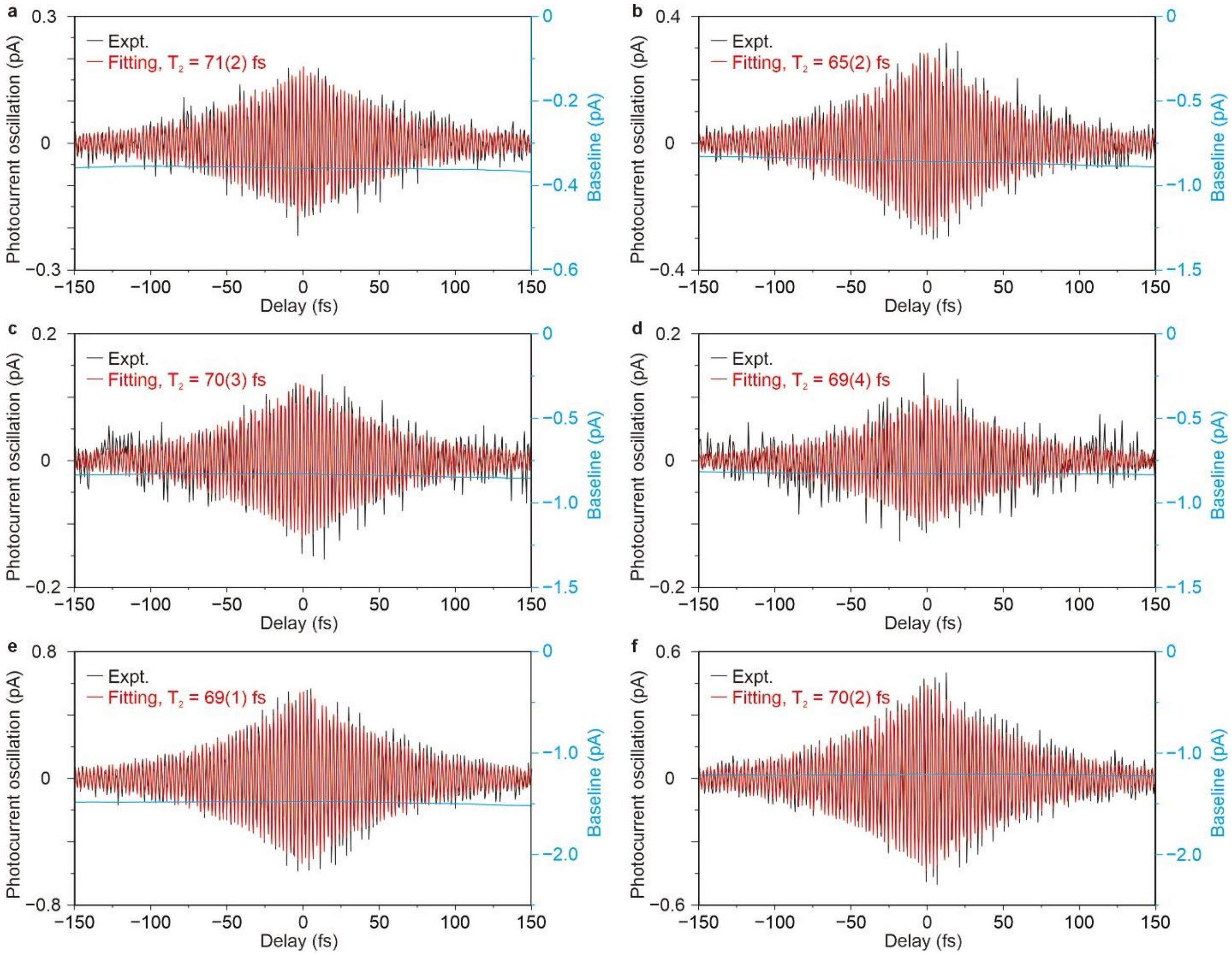

Supplementary Figure 10 | a-f: Time-resolved photocurrent traces measured as a function of the delay between pulse-1 and pulse-2 at six equidistant positions of the nanotip over the CuNc monomer (Fig. 5c, main-text). The oscillatory component (black curve) of the photocurrent was extracted by subtracting a smoothed baseline (blue curve) from the raw time trace. The FFT spectra of the time-resolved traces are shown in Fig. 5c (main-text). Measurement in panels **a** to **f** correspond one-to-one to the FFT spectra from top to bottom positons of the nanotip over the molecule. The decoherence time as well as the spectral position of the excitons is virtually insensitive to the lateral position of the nanotip over the molecules.

Density Functional Theory (DFT) and Time-Dependent Density Functional Theory (TDDFT) simulations

Atomistic calculations have been performed with the Gaussian 16 program package [1]. We carried out unrestricted all-electron calculations using the B3LYP functional [2] and the 6-31G(d,p) basis function, including the Grimme D3 [3] van der Waals correction (both in the dimer and in the monomer calculations for consistency). The neutral isolated Copper Naphthalocyanine (CuNc) molecule in its ground state has an unpaired electron, hence for the dimer CuNc calculation we investigated both the singlet and the triplet spin multiplicities; the triplet spin multiplicity turned out to be slightly favored in energy and was the one chosen for further analyses. The electronic excitation spectrum and transition dipole moments were calculated within a TDDFT approach [4,5] at the 6-31G-B3LYP level, obtaining up to the 30th excited state for the monomer and the 50th for the dimer. The transition densities were obtained using the Multiwfn post processing program [6]. The molecular structures and graphical representations of the molecular orbitals, transition densities, and transition dipole densities were prepared using GaussView 6.1.1 [7].

CuNc monomer DFT and TDDFT results

In Supplementary Figure 11, we report the frontier orbitals (HOMO and LUMO) for the CuNc monomer. Since the electronic multiplicity of the molecule is a doublet, we have different energies for the spin up and spin down molecular orbitals (MOs). The spatial wave functions of the corresponding orbital are identical in practice. Interestingly, there are two couples of degenerate LUMOs for both spin orientations. We observe that the calculated HOMO-LUMO gap is 1.872 eV for the spin up orbitals and 1.907 eV for the spin down orbitals, in excellent agreement with previously reported calculations [8]. The singly occupied molecular orbital (SOMO) is found at -6.176 eV, well below the HOMO orbital in the "A" list. The singly unoccupied molecular orbital (SUMO) is found at -1.934 eV, above the degenerate LUMO orbitals in the "B" list. A graphical representation of the SOMO and SUMO orbitals is given in Supplementary Figure 12. In both cases, the orbitals are centered on the Cu atom and their spatial extension can be traced back to a *d* atomic orbital, which spills out on the rest of the molecule.

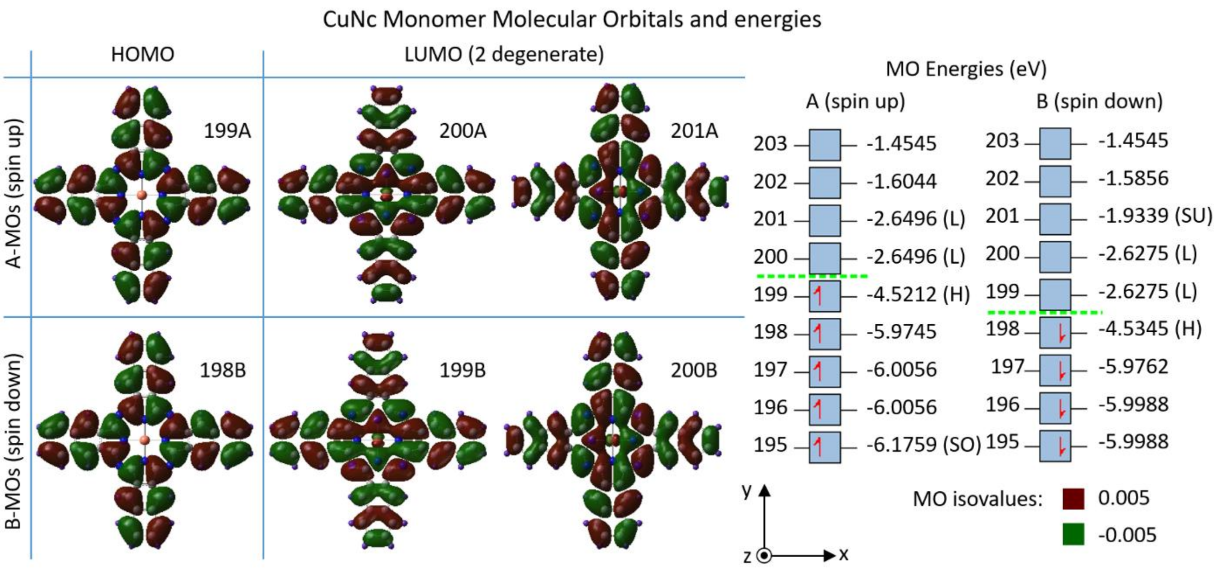

Supplementary Figure 11 | Frontier MOs and orbital energies of the CuNc monomer. Only the top view is given. All the shown orbitals are odd upon reflection over the molecular plane (z=0), which is a nodal plane. The orbitals are ordered in energy and assigned a cardinal number from lowest to highest and a letter, for which we follow the convention A=spin up and B=spin down. In the MO energy diagram, for each spin orientation, the HOMO and the two degenerate LUMOs are singled out by an “H” and “L”, respectively; the SOMO and SUMO are identified by “SO” and “SU”, respectively. In the bottom right corner, we give the Cartesian axes orientation used in the MO picture and the color code.

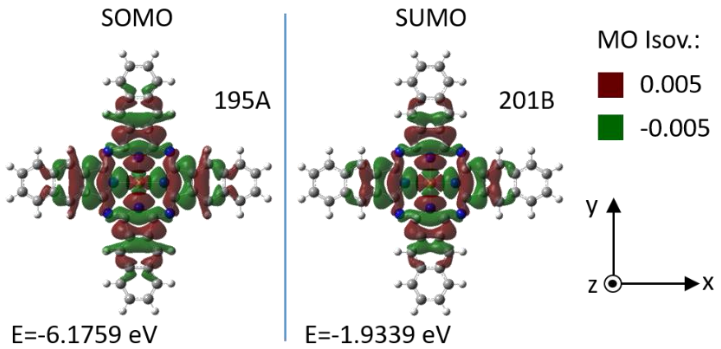

Supplementary Figure 12 | SOMO and SUMO orbitals of the CuNc monomer. Only the top view is given. Both orbitals are even upon reflection over the molecular plane (z=0), in which they take on a finite nonzero value.

Supplementary Table 1: CuNc monomer excited states as obtained by TD-DFT

State	Spin mult.	Energy (eV)	Energy (nm)	Transition dipole moment **d**(au)			Oscillator strength	
				d_x	d_y	d_z		
1	3.465	0.9277	1336	0	-0.0797	0	0.0001	
2	3.465	0.9277	1336	-0.0803	0	0	0.0001	
**3**	**2.003**	**1.7831**	**695**	**0**	**-3.6281**	**0**	**0.5750**	**E0**

**4**	**2.003**	**1.783**	**695**	**-3.6275**	**0**	**0**	**0.5748**	**E0**
5	2.006	1.8478	671	0	0	0	0	
6	3.465	2.0475	606	0	0	0	0	
7	3.465	2.1694	572	0	0	0	0	
8	3.465	2.2775	544	0	-0.0555	0	0.0002	
9	3.465	2.2775	544	-0.0557	0	0	0.0002	
10	2.028	2.4886	498	0	0	0	0	

In Table S1 we report the first 10 excited states of the CuNc monomer as obtained in a TDDFT calculation. In all cases we report the total spin multiplicity of the excited state and the energy (in eV and the corresponding conversion to nm). We also report the transition dipole moment, in atomic units (au), and the oscillator strength of the ground to excited state electronic transitions. We observe that, since the spin multiplicity of the ground state is 2, only the excited states with spin multiplicity 2 are reached upon the absorption of a photon. In the list given in Table S1 there are three such states (excited states 3, 4, and 5) that meet this criterion and have an energy comparable with the experimentally measured electronic transition. A lot of information is obtained by looking at the main MO transitions that give rise to each excited state. We found:

$$\Psi_{ES3} = 0.68854(|199A\rangle \rightarrow |201A\rangle) + 0.71299(|198B\rangle \rightarrow |200B\rangle), \quad (1)$$

$$\Psi_{ES4} = 0.68854(|199A\rangle \rightarrow |200A\rangle) + 0.71299(|198B\rangle \rightarrow |199B\rangle), \quad (2)$$

$$\Psi_{ES5} = |198B\rangle \rightarrow |201B\rangle. \quad (3)$$

Using as an example eq.1, the notation indicates that there are two main contributions to excited state 3, which is then written as a linear combination of two Slater determinants: the first (first term in eq. 1) is obtained from the ground state removing the electron from the 199A spin-orbital and putting it in the 201A spin-orbital (with an expansion coefficient 0.689); the second Slater determinant is obtained removing the electron from the 198B spin-orbital and putting it in the 200B spin-orbital (with a coefficient 0.713). The excited states 3 and 4 are a combination of HOMO-LUMO transitions. Given the symmetries of the HOMO and LUMO orbitals, these excited states have nonzero transition dipole moment for the in-plane coordinates (either d_x or d_y), while the out-of-plane transition dipole moment d_z vanishes [9]. Interestingly, the excited state 5 is a HOMO-LUMO+1 (actually it is a HOMO-SUMO) transition, which is dark and has a zero transition dipole moment for symmetry considerations. In our analysis we then selected the excited states 3 and 4 of the monomer, which are identified by the E0 label in table 1 and in the main text.

CuNc dimer DFT and TDDFT results

Upon the formation of a dimer of CuNc molecules the intermolecular interaction causes the formation of closely spaced frontier orbitals, derived from the parent HOMO and LUMO of the isolated CuNc molecule. This fact is made manifest in Figs. S13 and S14, where we report the frontier orbitals of a CuNc dimer in a triplet electronic configuration for the spin up (Supplementary Figure 13) and spin down (Supplementary Figure 14). For both spin orientations we find a “HOMO group” of two almost degenerate MOs, and a “LUMO group” made of four

almost degenerate MOs. Also in this case, while the orbital energies differ between spin up and spin down, the spatial shape of the MO is indistinguishable between the corresponding orbitals. Furthermore, it is possible to identify two occupied molecular orbitals in the A list, which result from combinations of the SOMOs of the monomer, and two unoccupied molecular orbitals in the B-list, which result from combinations of the SUMOs of the monomer. Additional information on these SOMO-derived and SUMO-derived orbitals is collected in Supplementary Figure 15.

As for the excited states of the dimer, we report in Table S2 the results for the lowest 20 excited states. Since the ground state is a triplet, only those states that have triplet spin multiplicity are accessible through light absorption. We further point out that there are six excited states (states 13 to 18) with triplet multiplicity in the 1.5 – 2.0 eV energy range. The expansion of the excited states 13 to 18 is given in eqs.4-9.

$$\begin{aligned}\Psi_{ES13} = {} & 0.45177(|397A\rangle \rightarrow |399A\rangle) + 0.18226(|397A\rangle \rightarrow |401A\rangle) + \\ & 0.47321(|398A\rangle \rightarrow |400A\rangle) + 0.11474(|398A\rangle \rightarrow |402A\rangle) + \\ & 0.46890(|395B\rangle \rightarrow |397B\rangle) + 0.18886(|395B\rangle \rightarrow |399B\rangle) + \\ & 0.49116(|396B\rangle \rightarrow |398B\rangle) + 0.11782(|396B\rangle \rightarrow |400B\rangle),\end{aligned} \tag{4}$$

$$\begin{aligned}\Psi_{ES14} = {} & -0.11278(|397A\rangle \rightarrow |400A\rangle) + 0.47827(|397A\rangle \rightarrow |402A\rangle) + \\ & -0.17398(|398A\rangle \rightarrow |399A\rangle) + 0.45002(|398A\rangle \rightarrow |401A\rangle) + \\ & -0.11803(|395B\rangle \rightarrow |398B\rangle) + 0.49981(|395B\rangle \rightarrow |400B\rangle) + \\ & -0.17738(|396B\rangle \rightarrow |397B\rangle) + 0.46320(|396B\rangle \rightarrow |399B\rangle),\end{aligned} \tag{5}$$

$$\begin{aligned}\Psi_{ES15} = {} & -0.18034(|397A\rangle \rightarrow |399A\rangle) + 0.45026(|397A\rangle \rightarrow |401A\rangle) + \\ & -0.11599(|398A\rangle \rightarrow |400A\rangle) + 0.47419(|398A\rangle \rightarrow |402A\rangle) + \\ & -0.18542(|395B\rangle \rightarrow |397B\rangle) + 0.46554(|395B\rangle \rightarrow |399B\rangle) + \\ & -0.11976(|396B\rangle \rightarrow |398B\rangle) + 0.49164(|396B\rangle \rightarrow |400B\rangle),\end{aligned} \tag{6}$$

$$\begin{aligned}\Psi_{ES16} = {} & 0.47412(|397A\rangle \rightarrow |400A\rangle) + 0.11103(|397A\rangle \rightarrow |402A\rangle) + \\ & 0.45286(|398A\rangle \rightarrow |399A\rangle) + 0.17610(|398A\rangle \rightarrow |401A\rangle) + \\ & 0.49068(|395B\rangle \rightarrow |398B\rangle) + 0.11477(|395B\rangle \rightarrow |400B\rangle) + \\ & 0.46872(|396B\rangle \rightarrow |397B\rangle) + 0.18084(|396B\rangle \rightarrow |399B\rangle),\end{aligned} \tag{7}$$

$$\begin{aligned}\Psi_{ES17} = {} & -0.49854(|395B\rangle \rightarrow |401B\rangle) - 0.49420(|395B\rangle \rightarrow |402B\rangle) + \\ & 0.50845(|396B\rangle \rightarrow |401B\rangle) + 0.49028(|396B\rangle \rightarrow |402B\rangle),\end{aligned} \tag{8}$$

$$\begin{aligned}\Psi_{ES18} = {} & -0.49721(|395B\rangle \rightarrow |401B\rangle) + 0.50136(|395B\rangle \rightarrow |402B\rangle) + \\ & -0.48752(|396B\rangle \rightarrow |401B\rangle) + 0.50538(|396B\rangle \rightarrow |402B\rangle).\end{aligned} \tag{9}$$

If we follow the convention to consider as HOMO the MOs belonging to the HOMO group of the dimer and as LUMO the MOs belonging to the LUMO group, then a quick inspection of ES17 and ES18 demonstrates that they are not formed by HOMO-LUMO transitions (in fact they are formed by HOMO-SUMO-derived transitions). For this reason, we focus on ES13, ES14, ES15, and ES16, which are all obtained as linear combinations of HOMO-LUMO transitions. In Table S2, they are identified as E1, E2, E3, and E4, as in the main text. We observe that these four excited states are derived from the two E0 excited states of the monomer. The degeneracy is lifted by the

intermolecular interaction so that they are split into 4 distinct states. Interestingly, while all of them are characterized by d_z=0 (a property derived from the "parent" E0 states), only E1 and E3 have nonzero in-plane transition dipole moments. As consequence, E1 and E3 can be populated via light absorption, whereas E2 and E4 are "dark" states (they have zero oscillator strength).

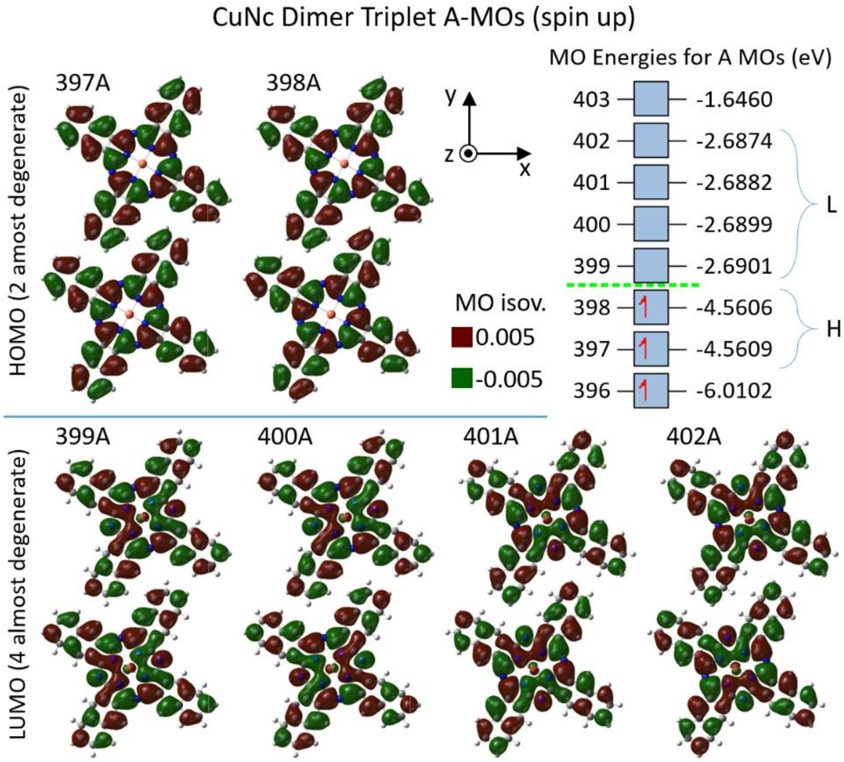

Supplementary Figure 13 | Frontier MOs and orbital energies of the CuNc dimer for the spin up MOs. All the shown orbitals are odd upon reflection over the molecular plane (z=0), which is a nodal plane. Only the top view is given. In the MO energy diagram, the HOMO group (H) and the LUMO group (L) are highlighted.

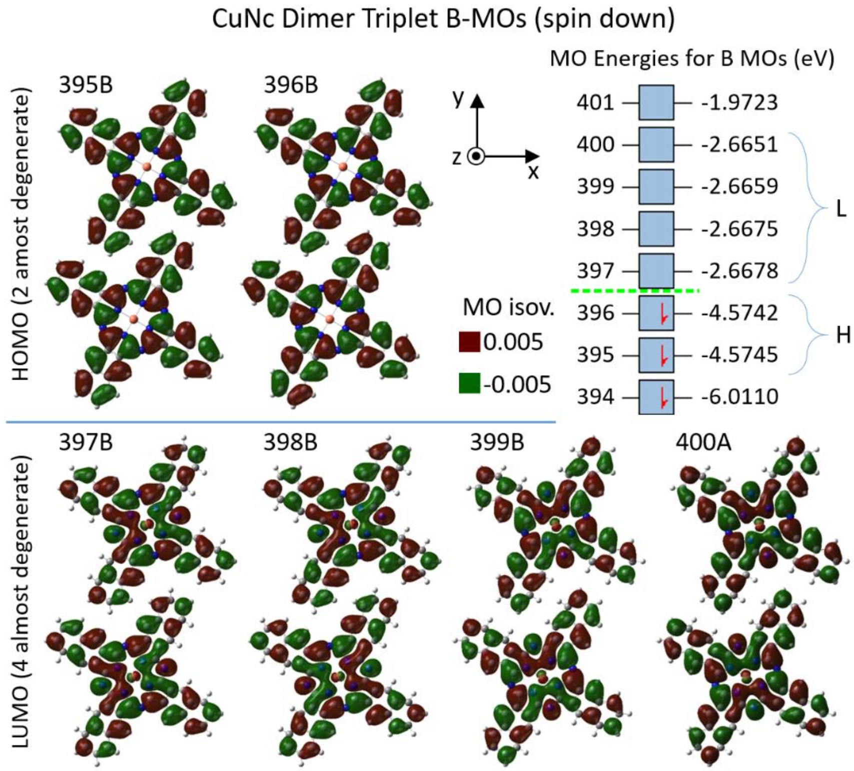

Supplementary Figure 14 | Frontier MOs and orbital energies of the CuNc dimer for the spin down MOs. Same as for Fig.S6.

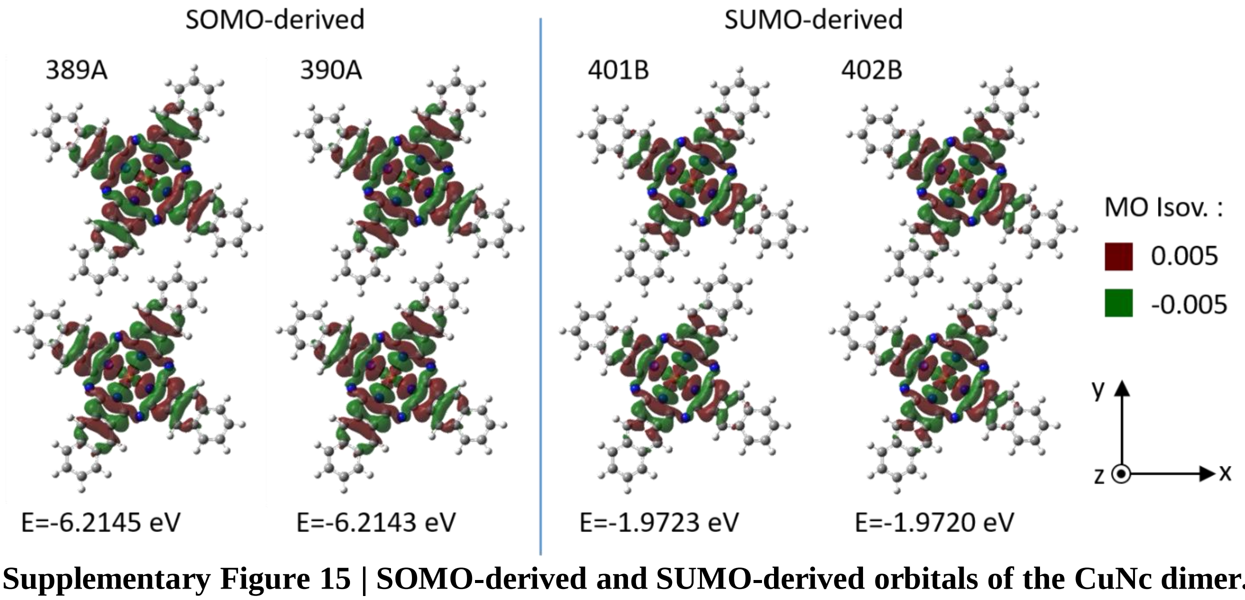

Supplementary Figure 15 | SOMO-derived and SUMO-derived orbitals of the CuNc dimer. Only the top view is given. All orbitals are even upon reflection over the molecular plane (z=0), in which they take on a finite nonzero value.

Supplementary Table 2: CuNc dimer excited state as obtained by TD-DFT

State	Spin mult.	Energy (eV)	Energy (nm)	Transition dipole moment (au)			Oscillator strength	
				d_x	d_y	d_z		
1	4.125	0.9262	1339	-0.0261	0.1259	0	0.0004	
2	4.125	0.9262	1339	-0.0043	0.0209	0	0	
3	4.125	0.9296	1334	0.0242	0.008	0	0.003	
4	4.125	0.9296	1334	0.1003	0.0332	0	0	
5	3.608	1.6812	738	-0.0013	0.0016	0	0	
6	3.608	1.6812	738	-0.0008	0.001	0	0	
7	3.608	1.6869	735	0.0056	-0.0042	0	0	
8	3.608	1.6869	735	-0.0084	0.0063	0	0	
9	3.608	1.7171	722	-0.0036	0.0036	0	0	
10	3.608	1.7171	722	0.0024	-0.0024	0	0	
11	3.609	1.7228	720	0.0054	-0.0054	0	0	
12	3.608	1.7228	720	-0.0145	0.0145	0	0	
**13**	**3.004**	**1.7497**	**709**	**0.2234**	**5.7351**	**0**	**1.4121**	**E1**
**14**	**3.004**	**1.7629**	**703**	**0.0002**	**-0.0002**	**0**	**0.0**	**E2**
**15**	**3.004**	**1.7967**	**690**	**4.8584**	**-0.2200**	**0**	**1.0411**	**E3**
**16**	**3.004**	**1.8056**	**687**	**-0.0011**	**0.0003**	**0**	**0.0**	**E4**
17	3.006	1.8489	671	0	0	0	0	
18	3.006	1.8489	671	0	0	0	0	
19	4.125	2.0464	606	0.0011	-0.0041	0	0	
20	4.125	2.0464	606	0.0003	-0.0011	0	0	

Transition Density

The transition density between the initial state Ψ_i and the final state Ψ_j gives a microscopic vision of the electron transfer upon the electronic transition. It is defined as:

$$\rho^{ij}(\boldsymbol{r}) = N\int_{\Omega}\Psi_i^*(\boldsymbol{r},\boldsymbol{r}_2..\boldsymbol{r}_N)\Psi_j(\boldsymbol{r},\boldsymbol{r}_2..\boldsymbol{r}_N)d^3r_2...d_N^3, \quad (10)$$

where N is the number of electrons in the system and Ω is the total volume. In our case, the initial state is the ground state, whereas the final state is one of the relevant excited states of either the CuNc monomer or the CuNc dimer. In this way, we can identify and plot the transition density associated to a given excited state, which, dropping the initial state index, is indicated as ρ^j. If one assumes that the excited state Ψ_j can be written as an expansion of M single electron excitations in which an electron is transferred from one spin orbital in the ground state $|g\rangle$ to a spin orbital in the excited state $|e\rangle$, (eqs. 1-9), i. e.

$$\Psi_j = \sum_{l=1}^{M} c_l^j(|g_l\rangle \rightarrow |e_l\rangle), \quad (11)$$

equation (10) is simplified via the orto-normality relation of the spin orbitals, yielding:

$$\rho^j(x,y,z) = \sum_{l=1}^{M} c_l^j \; \varphi_{g_l}^*(x,y,z) \;\; \varphi_{e_l}(x,y,z). \tag{12}$$

where $\varphi_n(x,y,z)$ denotes the spatial function associated to the $|n\rangle$ spin orbital. Equation 12 is readily evaluated, known the expansion coefficients c_l^j, and the spatial form of the spin orbitals φ. In our cases we obtained the spin orbitals on a spatial grid (spacing $\frac{1}{6}$ au) using the cubegen utility within the Gaussian package and the transition density on the same spatial grid via the Multiwfn post processing tool.

In Supplementary Figure 16 we report the transition density for the excited states 3 and 4 of the monomer, associated to the E0 feature in the monomer spectra. We note for both excited states, ρ^j is an even function of the z coordinate and vanishes for z=0. This property is directly derived from the HOMO-LUMO spin orbitals, which are the transition that mainly contribute to these excited states. This symmetry causes that both states have a vanishing component of the out-of-plane transition dipole matrix element. The same symmetry property (not explicitly shown) is found for the E1-E4 states of the dimer.

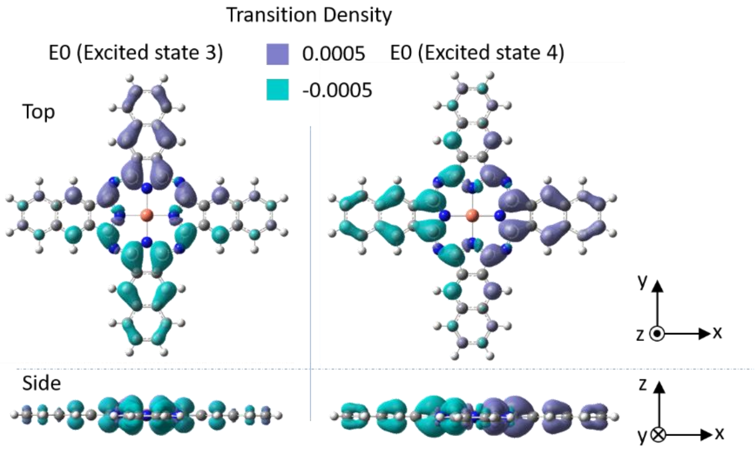

Supplementary Figure 16 | Transition density of the relevant excited state in the monomer. Spatial variation (top view and side view) of the transition density for the 3rd and 4th excited states (E0) of the CuNc molecule. In both cases, ρ^j is even for reflections on the molecular plane ($z \rightarrow -z$), even though it vanishes at $z = 0$.

Two point-dipole modelling of the electric potential in the STM nanocavity

A simple model for the electric field in the STM cavity assumes it is generated by two point dipoles [9]: one located at the STM tip and the other at its image position on the metal surface. Both dipoles are assumed to be aligned in the same direction, perpendicular to the xy-plane, which is parallel to the supporting surface and the molecular plane. In these conditions, the electrostatic potential Φ in a given point (x,y,z) is given by:

$$\Phi(x,y,z) = \frac{p\,(z-z_T)}{((x-x_T)^2+(y-y_T)^2+(z-z_T)^2)^{1/2}} + \frac{p\,(z-z_S)}{((x-x_T)^2+(y-y_T)^2+(z-z_S)^2)^{1/2}}, \tag{11}$$

In eq.11 p represents the intensity of the dipole (p is a common term and accounts for a multiplicative factor only), x_T y_T, and z_T are the cartesian components of the tip position, whereas

z_S is the vertical position of the point dipole in the metal surface (notice that we are assuming that the in-plane position of the point dipole in the metal surface is equal to the ones of the tip, i. e. $x_S = x_T$ and $y_S = y_T$). Since the surface point dipole is located at the image position of the tip inside the surface, z_S is in principle determined, once z_T is set, knowing the number of NaCl layers deposited on the surface.

A microscopic model of the potential within the plasmonic cavity, allows to estimate the coupling of the light with the excited state and define an effective oscillator strength:

$$\tilde{f}^j \propto \left| \int_{-\infty}^{\infty} \int_{-\infty}^{\infty} \int_{-\infty}^{\infty} \rho^j(x,y,z) \cdot \Phi(x,y,z) \; dx\, dy\, dz \right|^2 \tag{12}$$

In this work we have set the origin of the z axis on the molecular plane and used $z_T = 11$ au and $z_S = -56$ au, which account for a total of 3 NaCl layer on the metallic surface, and are consistent with the values used in the work by Jaculbia et al. [9].

It is important to point out that we have carried out several tests, varying the z_T and z_S input values and finding qualitatively similar results as the ones reported here and in the main text, provided that the vertical tip position was set to $z_T \geq 10$ au. We conclude this section observing that Φ, as given in eq.11 diverges at $\{x,y,z\} = \{x_T, y_T, z_T\}$ and $\{x,y,z\} = \{x_T, y_T, z_S\}$. The latter point, being far away from the molecular plane, is never included in the spatial grid used to integrate eq. 18. As for the divergence in $\{x_T, y_T, z_T\}$, we have verified that all the spin orbital used for the computations in the expansion of the relevant exited states (eqs.1-9) vanish for $z > 10$ au, and so does the transition density.

Supplementary References

[1] M. J. Frish et al, Gaussian 16, Revision C.01, Gaussian Inc., Wallingford CT (2919).
[2] A. D. Becke, Density-functional thermochemistry. III. The role of exact exchange. J. Chem. Phys. **98**, 5648–5652 (1993).
[3] S. Grimme, J. Antony, S. Ehrlich, H. Krieg, A consistent and accurate ab initio parametrization of density functional dispersion correction (DFT-D) for the 94 elements H-Pu, J. Chem. Phys., **132**, 154104 (2010).
[4] E. Runge, E. K. U. Gross, Density-functional theory for time-dependent systems. Phys. Rev. Lett. **52**, 997–1000 (1984).
[5] M. E. Casida, Recent advances in density functional methods part I (ed. Chong, D. P.) 155–192 (1995).
[6] Tian Lu; A comprehensive electron wavefunction analysis toolbox for chemists, Multiwfn. J. Chem. Phys. **161** 082503 (2024).
[7] R. Dennington, T. Keith, J. Millam, Gaussview, Version 6.1.1, Semichem Inc., Shawnee Mission, KS (2019).
[8] Zhongqiang Liu, Zhao-Xu Chen, Biaobing Jin, Density functional theory studies on the structures and vibrational spectroscopic characteristics of nickel, copper and zinc naphthalocyanines, Spectrochimica Acta Part A: Molecular and Biomolecular Spectroscopy, **217**, 8 (2019).

[9] R. B. Jaculbia, H. Imada, K. Miwa, T. Iwasa, M. Takenaka, B. Yang, E. Kazuma, N. Hayazawa, T. Taketsugu, Y. Kim Single-molecule resonance Raman effect in a plasmonic nanocavity, Nature Nanotechnology **15**, 105 (2020).

[10] Tomáš Neuman, Ruben Esteban, David Casanova, Francisco J. García-Vidal, and Javier Aizpurua, Coupling of Molecular Emitters and Plasmonic Cavities beyond the Point-Dipole Approximation, Nano Letters **18**, 2358-2364 (2018)

[11] Ben Yang, Gong Chen, Atif Ghafoor, Yufan Zhang, Yao Zhang, Yang Zhang, Yi Luo, Jinlong Yang, Vahid Sandoghdar, Javier Aizpurua, Zhenchao Dong, and J. G. Hou, Sub-nanometre resolution in single-molecule photoluminescence imaging. Nat. Photonics **14**, 693–699 (2020)
