# Supplementary material for: Orbital-resolved imaging of coherent femtosecond exciton dynamics in coupled molecules
Source: Nat Commun. 2026 May 15;17:6455. doi: 10.1038/s41467-026-73191-0 (PMC13376558; doi:10.1038/s41467-026-73191-0)
Supplement: Supplementary file 2 — Transparent Peer Review file [file 41467_2026_73191_MOESM2_ESM.pdf]

nature portfolio

Peer Review File

Orbital-resolved imaging of coherent femtosecond exciton dynamics in coupled molecules

Corresponding Author: Dr Manish Garg

This file contains all reviewer reports in order by version, followed by all author rebuttals in order by version.

Attachments originally included by the reviewers as part of their assessment can be found at the end of this file.

Version 0:

Reviewer comments:

Reviewer #1

(Remarks to the Author)
The article entitled "Orbital-resolved imaging of coherent femtosecond exciton dynamics in coupled molecules" by Luo et al. is an experimental study, supported by theoretical calculations, investigating excited-state dynamics in molecules of Cu naphthalocyanine using the method of detecting photocurrents upon photoexciting the molecule. The authors perform autocorrelation measurements by applying a pair of laser pulses exciting the molecule at distinct times and then detecting the resulting photocurrent. By varying the time delay between the pulses, an oscillating component of the photocurrent is found that is attributed to the autocorrelation of the excitonic wave function. From this, the coherence properties of the exciton are obtained. This approach could potentially be useful for studies of real-time dynamics of molecular excited states and e.g. intermolecular energy transfer.

The experimental study is convincing, clearly presented and the interpretation of the results seems sound. The theory supporting the experiment, particularly the (TD)DFT calculations is less clearly presented and, in my opinion, should be amended (see below). Overall, I recommend the paper for publication, but only after major revision of the theory and clarifying other questions and comments below.

The interpretation of the result shown by the authors in terms of autocorrelation functions of the exciton state strongly suggests comparing their results to the optical absorption spectra of the molecule, which should be directly related to the same correlation function. My primary question would therefore be: what information, different from the one obtained from optical absorption spectroscopy, is obtained in this photocurrent measurement? Absorption (or action/excitation) spectroscopy could be performed in the STML conditions as well using tunable lasers (as demonstrated in Imada et al., Science 373(6550), 95-98, 2021) and likely follows the same optical selection rules as the present experiment (meaning that it is governed by the same plasmon-exciton coupling). I think that it is important to clearly answer this question to show the potential of the present autocorrelation technique.

When reading the manuscript, the fact that the molecule is in a doublet state seems important at first, but, in fact, the molecule behaves exactly as other phthalo/naphthalocyanines with closed-shell ground state. The unpaired electron seems to occupy a deeper lying orbital that does not participate in the formation of the excitation studied here nor the PIR and NIR. The authors should explicitly comment on this in the text and clarify the role of the unpaired electron in the experiment. They should also stress the diference from e.g. doublet emitters where SOMO and SUMO directly participate in the low-energy optical response. Similarly, for the dimer it does not really seem relevant if the ground state is triplet or open-shell singlet. Is that right? Please, discuss. Also, detailed analysis of the SUMO/SOMO should be presented in the SI similarly as it is done for HOMO and LUMO in Fig.S4. Right now the role and location of SOMO and SUMO is unclear.

I do not understand why the authors operate with the transition dipole (Eq.3) and then define the "space resolved version of the transition dipole matrix element" (below Eq.3) as mu_alpha(x,y,z)=psi^*_i alpha psi_j, with alpha being x, y, or z. The space resolved version of the transition dipole is the transition density (or transition current density, requiring different treatment), which can be straightforwardly obtained from quantum-chemical software. Transition density can be readily used for the evaluation of plasmon-exciton coupling using the model proposed in the SI by evaluating the quasi-static potential phi instead of the electric field in Supp. Eq.(19-21). The coupling is then obtained as an integral over transition density multiplied

by phi (that squared, to obtain the "oscillator strength"), rather than by Eq.(18). In fact, if I am not mistaken, the present theory, Eq.(18), is incorrect as it implies that the quasi-static potential of the plasmon can be obtained from the electric field as phi=x*Ex+y*Ey+z*Ez, which would be incorrect. I suggest that the authors use the well-accepted and easy-to-use theory employing the transition density. Alternatively, the authors can evaluate the transition current density and apply the appropriate theory. Correcting this point is critical, in my opinion.

Related to the comment above, I strongly suggest that in the Supp.Fig. S7,S8 and Fig.4 of the main text the transition density is shown. It is much more physically relevant and representative than the present plots as the transition density reveals the microscopic charge distribution (and hence dipoles). I discourage the authors from using the concept of the "differential form of the transition matrix element" as it is a poorly defined quantity depending on the choice of coordinates and brings limited insight.

In Fig. 2a the authors show the photocurrent that is slowly changing as bias voltage is applied. This does not seem like an abrupt change of the photocurrent when a threshold is passed, as suggested in Fig.2c,d. How do the authors explain this behavior? What mechanism is behind it? Does it have any implications for the interpretation? How does it relate to the previous photocurrent studies in STM on molecules that suggested that substrate reorganization stands behind gradual onsets?

In Fig.3 the comparison of the FFT of the autocorrelation function alongside with the electroluminescence spectrum. The latter is evidently much broader. How do the authors understand that? This is related to my previous question about comparing the FFT of the autocorrelation to the absorption spectrum.

Third paragraph of the section "Atomic-scale control of dark and bright triplet excitons": I do not understand why the observation of four spectral peaks for the coupled dimer implies site-dependent excitonic coupling (exciton-exciton coupling). It rather implies the well-known breaking of selection rules and the possibility to access the dark excitons as well. The authors should show full spectrally filtered (electroluminescence or photoluminescence...) photon maps at the energies of the observed peaks to reveal their origin. I also want to point out that the possibility to measure dark states has been well known in the community for almost a decade (as correctly cited by the authors).

I find the conclusions very generic and vague. What is the actual concrete finding of this study that is unique to it? How concretely is it relevant to light-harvesting complexes or singlet fission?

There is a typo in Supp. Eq.10

Reviewer #2

(Remarks to the Author)
In this manuscript entitled “Orbital-resolved imaging of coherent femtosecond exciton dynamics in coupled molecules”, Luo et al. report an impressive implementation of femtosecond wave-packet interferometry in an STM junction, enabling orbital-resolved mapping of excitonic decoherence at the single-molecule level. The authors further compare monomer and dimer cases to reveal the influence of intermolecular coupling on the coherence time. The modeling for the molecular transition dipoles and the tip-enhanced electric field is also qualitatively consistent with the experimental observations. The technical execution is outstanding, and the work represents a notable advance in ultrafast STM spectroscopy. I fully support its publication in Nature Communications, with only a few items that the author may consider addressing that might help the readers to better appreaciate this work.

The manuscript presents the measured coherence times for the monomer and dimer but does not sufficiently discuss the microscopic origin of the decoherence in the single-molecule limit. Is it mainly driven by electronic dephasing, vibronic coupling, substrate-induced fluctuations, or hybridization with the metallic environment? Since the authors show that reducing the NaCl thickness changes the coherence time, it would be helpful to clarify the relative roles of molecule–substrate coupling and to comment on possible strategies to tune T_2 using different insulating layers or substrates. It would also be useful to compare the observed values with known dephasing rates in related molecular systems.

Prior STM studies have shown that the local tip–sample electric field can shift molecular orbital energies and thereby modify excitonic transitions. In Fig. 5c, the slight peak shifts with lateral tip position may reflect such effects. The authors should clarify whether systematic tip-height or lateral-position dependence was observed, and whether the extracted exciton energy and T_2 remain consistent under such perturbations. It would also be useful to comment on whether adjusting the tunneling channel via bias affects the measured coherence. A quantitative analysis of peak energy versus tip position would strengthen the interpretation.

In Fig. S2b, a small shoulder appears on the low-energy side of the FFT spectrum with an amplitude of ~0.1, which is comparable to the E_4 peak of the dimer in Fig. 4b. The origin of this feature is unclear. The authors should comment on whether it arises from vibronic replicas, instrumental artifacts, or weak excited states, and whether it is reproducible over multiple measurements and molecules. Some clarification or control data are needed to rule out artifacts.

The dimer results show both energy splitting and a reduced coherence time. Have the authors investigated dimers with controlled intermolecular separations or different geometries? Such data would be valuable for understanding how coupling strength influences exciton delocalization and decoherence, and whether the bright/dark nature evolves gradually as the coupling is tuned. It would also be interesting to know whether charge-transfer excitonic character appears at smaller

separations.

The excitation bandwidth (1450–1900 meV) is broad enough to drive vibronic transitions and create multi-mode wave packets. How does this affect the extracted coherence times? Are vibrational coherences contributing to the dephasing, and could some of the FFT features originate from vibronic beating? A brief discussion or theoretical estimate separating electronic and vibronic contributions would help clarify this point.

Lastly, just a quick note: as a fellow STM researcher, I cannot agree more that phthalocyanine derivatives are a favorable system for proof-of-principle studies, particularly when demonstrating technical advancements such as those presented in this work. However, this is also a decades-old model system that has been extensively investigated from many perspectives. I would look forward to seeing more insights drawn from other molecular systems, which I expect could provide even more valuable and exciting chemical and physical understanding.

Reviewer #3

(Remarks to the Author)
I co-reviewed this manuscript with one of the reviewers who provided the listed reports. This is part of the Nature Communications initiative to facilitate training in peer review and to provide appropriate recognition for Early Career Researchers who co-review manuscripts.

Reviewer #4

(Remarks to the Author)
In this manuscript, the authors demonstrate real-space observation of coherent exciton dynamics using ultrafast STM. Specifically, a CuNc molecule adsorbed on an ultrathin NaCl layer within a plasmonic STM junction is excited by 15-fs visible laser pulses, and the resulting photon-induced tunneling current is measured with high precision to visualize the ultrafast exciton dynamics at the single-molecule level. Previous studies in similar systems have reported excitonic resonances and energy transfer under continuous-wave illumination, and more recently, ultrafast dynamics have been probed using THz-STM. However, to the best of my knowledge, this work represents the first demonstration of exciton dynamics in a single molecule driven and detected using few-cycle visible/near-infrared pulses. While ultrafast STM has previously been applied to coherent phonons in oxide thin films, it has not yet been extended to molecular excitons. Therefore, this study constitutes a significant and timely advancement toward ultrafast molecular spectroscopy using visible/near-infrared STM.
Ultrafast STM techniques that enable combined atomic-scale spatial resolution and femtosecond temporal resolution are rapidly advancing and are attracting growing attention due to their potential impact in quantum science and nanoscale condensed-matter research. The methodology demonstrated here is at the forefront of the field, the experimental data are of excellent quality, and the analysis, including first-principles calculations, is rigorous and thorough. The discussion of prior work is appropriate and the cited literature is relevant.
Based on these considerations, I believe this manuscript will attract broad interest within the nanoscience and nanotechnology community, and its scientific merit supports publication in Nature Communications.

Minor comments:
1. The observed excitonic response may depend on the tip–molecule distance, for example through tip-induced Stark shifts, tip-induced orbital hybridization, or modifications to the exciton dephasing time arising from tip–molecule interactions. Although this does not affect the main conclusions, a brief discussion on this point would strengthen the manuscript.
2. The strong sensitivity achieved in this work is likely supported by the localized surface plasmon resonance (LSPR) formed in the STM junction. At the same time, the enhanced optical near-field may also influence the excitonic response and photocurrent generation. While this does not alter the conclusions, I encourage the authors to include a brief discussion on the possible influence of LSPR, and, if feasible, present the STML spectrum to show the optical response of the plasmonic junction.
3. The plasmonic field enhancement and photocurrent sensitivity in STM-based optical measurements can significantly depend on the exact tip geometry and material condition. It would be helpful if the authors could comment on the reproducibility across multiple STM tips.
4. Given the intense electric field associated with the LSPR and 15 fs laser pulses, there may be concerns regarding laser-induced tip or molecule modification, heating, or photo-desorption effects. It would be helpful if the authors could clarify whether any potential contributions of these effects.
5. Since the photocurrent oscillations are used as a proxy to reconstruct exciton coherence, it may be useful to specify whether the pulse duration quoted (~15 fs) refers to the free-space pulse or the effective pulse duration in the plasmonic junction. If the latter is unknown, a short clarification acknowledging that plasmonic filtering may modify spectral phase would improve clarity.
6. In some sentences, the term “exciton” appears to refer broadly to any excited-state transient wavepacket, whereas in others it refers to a spectroscopically defined bound state. I suggest ensuring consistent terminology or defining usage early in the manuscript.
7. Since the electronic structure of a molecule is determined by its wavefunction, the term local electronic distribution may be more appropriate than electronic inhomogeneity.
8. The manuscript states that “A photocurrent of ~100 fA at an 80-MHz repetition rate corresponds to ~0.008 electrons per laser pulse”. However, because STM detects only the DC component of the current, the AC component induced by photoexcitation may not be directly measurable. In that regard, clarification on whether this estimate represents a physically

meaningful quantity would be appreciated.

Version 1:

Reviewer comments:

Reviewer #1

(Remarks to the Author)
The authors have revised the paper and reacted to the comments of the reviewers. I still believe that the paper is of high experimental quality and deserves publication.

In response to my comments the authors removed a part of the supplementary material concerning the microscopic details of the plasmon-exciton interaction and introduced a new simplified way to treat the local interactions. Unfortunately, I think that my comments were not understood by the authors and their modifications concerning the use of the differential dipole density ("transition dipole density") \mu and its use to describe the localized plasmon-exciton interaction in Supp. Eq. 18 made the theory even less physically meaningful. In a separate document, I therefore attach a brief description of the technical problems that I see with the approach of the authors as well as a suggestion how to correct it. This issue concerns both the main text (Rev#2) and, primarily, the Supp. Mat.

The authors are right that "transition dipole density" can be defined and is accessible in quantum chmistry codes. However, this does not have any implications regarding its physical meaning, particularly in the context of STM. The excistence of literature actually using the "transition dipole density" by no means shows that its use is appropriate, certainly not in the present context.

To be clear, I don't think that the use of the "transition dipole density" is a matter of taste, I am convinced that, when dealing with inhomogeneous plasmonic field, it is strictly incorrect (in the mathematical sense). I therefore cannot recmmend the paper for publication unless this issue is satisfactorily resolved (for the sake of the quality of the text and preventing the spread of misconceptions). If the authors show me a rigorous mathematical proof (i.e. not just a reference to external resources) that the approach using "transition dipole density" is correct in the present context of inhomogeneous plasmonic field, I will be happy to accept its use. Otherwise I must insist on using a different, physically meaningful, approach.

I appreciate the additions to the discussion of the open-shell character of the molecule and the triplet character of the dimer. I also appreciate the addition of transition densities in the Supp. Mat.

Regarding my question on how abrupt/smooth the changes in photocurrent with external bias are: I am not sure if the authors fully appreciate the references that are cited in the answer (Nature 603, 829–834 (2022); Nature Communications 16 (1), 6039 (2025)). If the photocurrents simply came from the relative alignmnent of the electronic levels of the molecule with the Fermi level of the electrodes, one would expect sharp variations of the photocurrents upon reaching certain theresholds. As far as I understand, it is the substrate reorganization that smears out these thresholds and thus enables the fine-tuning of the balance between various photocurrent channels (related to the phonon-blockade effect). Maybe the authors could comment on this effect in the main text as well, as this is a subtle, but important, detail that could improve the awareness of the reader and prevent the reader from falling for misconceptions.

The addition of rev#3 is appreciated.

There are typos in Eq. 3 of the main text and Supplementary Eq. 10, 11. I also ask the authors to check for the presence of factors of "N" in these equations. I suspect that it might be missing (depending on how the quantities are normalized).

Page 6 of the main text: SOMO and SUMO orbitals -> SUMO and SUMO (O stands for orbital).

Reviewer #2

(Remarks to the Author)
The author has addressed my concerns satisfactorily. I suggest including some (if not all) of the discussion in the response into SI or manuscript as they are highly valuable for readers to better appreciate the content.

Reviewer #3

(Remarks to the Author)
I co-reviewed this manuscript with one of the reviewers who provided the listed reports. This is part of the Nature Communications initiative to facilitate training in peer review and to provide appropriate recognition for Early Career Researchers who co-review manuscripts.

Reviewer #4

(Remarks to the Author)

The authors have addressed my comments and those of the other referees in a thorough and sincere manner, supported by substantial new data and clarifications. Importantly, they provide additional experimental evidence, which significantly strengthens the reproducibility and interpretation of the results.

The theoretical description has also been meaningfully improved, including correction of problematic aspects of the field modeling and the addition of clearer supporting analysis in the Supplementary Information.

Overall, the revisions are convincing, and the responses satisfactorily resolve the major concerns. I therefore recommend publication of the manuscript in Nature Communications.

Open Access This Peer Review File is licensed under a Creative Commons Attribution 4.0 International License, which permits use, sharing, adaptation, distribution and reproduction in any medium or format, as long as you give appropriate credit to the original author(s) and the source, provide a link to the Creative Commons license, and indicate if changes were made.
In cases where reviewers are anonymous, credit should be given to 'Anonymous Referee' and the source.
The images or other third party material in this Peer Review File are included in the article's Creative Commons license, unless indicated otherwise in a credit line to the material. If material is not included in the article's Creative Commons license and your intended use is not permitted by statutory regulation or exceeds the permitted use, you will need to obtain permission directly from the copyright holder.
To view a copy of this license, visit https://creativecommons.org/licenses/by/4.0/

Response to the referees

We would like to cordially thank all the referees for their invaluable comments. We are happy to read that all the reviewers appreciated our manuscript, and judged it "convincing and clearly presented" (**reviewer #1**), "impressive implementation of femtosecond wave-packet interferometry in an STM junction" (**reviewer #2**), and "significant and timely advancement toward ultrafast molecular spectroscopy using visible/near-infrared STM'' (**reviewer #4**). We have revised the manuscript in accordance with their suggestions and criticisms, which all have been fully addressed. We mark essential revisions as **REV#** and have underlined the new text (____) in the main text and supplementary materials (SM) to ease the evaluation.

We hope that the referees will acknowledge the significant improvements made in the manuscript, certainly driven by their keen comments, and will now be happy to recommend it for publication in Nature Communications. Below we enlist detailed responses to all comments of the referees.

REVIEWER COMMENTS

Reviewer #1 (Remarks to the Author):

The article entitled "Orbital-resolved imaging of coherent femtosecond exciton dynamics in coupled molecules" by Luo et al. is an experimental study, supported by theoretical calculations, investigating excited-state dynamics in molecules of Cu naphthalocyanine using the method of detecting photocurrents upon photoexciting the molecule. The authors perform autocorrelation measurements by applying a pair of laser pulses exciting the molecule at distinct times and then detecting the resulting photocurrent. By varying the time delay between the pulses, an oscillating component of the photocurrent is found that is attributed to the autocorrelation of the excitonic wave function. From this, the coherence properties of the exciton are obtained. This approach could potentially be useful for studies of real-time dynamics of molecular excited states and e.g. intermolecular energy transfer.

The experimental study is convincing, clearly presented and the interpretation of the results seems sound. The theory supporting the experiment, particularly the (TD)DFT calculations is less clearly presented and, in my opinion, should be amended (see below). Overall, I recommend the paper for publication, but only after major revision of the theory and clarifying other questions and comments below.

We would like to sincerely thank the reviewer for their kind words and for the thorough evaluation of our manuscript. We have made all possible efforts to improve the clarity of all the points raised and to meet the expectations of the reviewer. Below, we provide a point-by-point response to the comments raised.

The interpretation of the result shown by the authors in terms of autocorrelation functions of the exciton state strongly suggests comparing their results to the optical absorption spectra of the molecule, which should be directly related to the same correlation function. My primary question

would therefore be: what information, different from the one obtained from optical absorption spectroscopy, is obtained in this photocurrent measurement? Absorption (or action/excitation) spectroscopy could be performed in the STML conditions as well using tunable lasers (as demonstrated in Imada et al., Science 373(6550), 95-98, 2021) and likely follows the same optical selection rules as the present experiment (meaning that it is governed by the same plasmon-exciton coupling). I think that it is important to clearly answer this question to show the potential of the present autocorrelation technique.

We thank the reviewer for this comment. While absorption and emission spectroscopies can indeed capture the energy hierarchy and the associated line shapes, nonetheless, they offer only indirect access to real-time dynamics, for instance, through spectral lineshape analysis. In contrast, the technique demonstrated in the current work ports the powerful approach of wavepacket interferometry to single molecules in the STM junction, which provides a direct measurement of molecular excitonic dynamics.

Utilizing this approach, we have captured the exciton dephasing time in single CuNc molecules, and investigated the influence of intermolecular interactions. Owing to such interactions, excitons dephase faster in interacting molecules, compared to isolated single molecules (T2 ~ 70 fs). It is worth mentioning that in interacting molecules, the dephasing time is electronic state dependent (please see Fig. S8 in SI). This direct quantification of decoherence and its dependence on intermolecular interactions is inaccessible through steady-state, frequency-domain spectroscopic approaches alone. The capability of the wavepacket interferometric approach will be further advanced to investigate dynamics in single molecules (e.g. energy transfer dynamics, conical intersections etc.) in future works.

When reading the manuscript, the fact that the molecule is in a doublet state seems important at first, but, in fact, the molecule behaves exactly as other phthalo/naphthalocyanines with closed-shell ground state. The unpaired electron seems to occupy a deeper lying orbital that does not participate in the formation of the excitation studied here nor the PIR and NIR. The authors should explicitly comment on this in the text and clarify the role of the unpaired electron in the experiment. They should also stress the diference from e.g. doublet emitters where SOMO and SUMO directly participate in the low-energy optical response. Similarly, for the dimer it does not really seem relevant if the ground state is triplet or open-shell singlet. Is that right? Please, discuss. Also, detailed analysis of the SUMO/SOMO should be presented in the SI similarly as it is done for HOMO and LUMO in Fig.S4. Right now the role and location of SOMO and SUMO is unclear.

The reviewer is right: the unpaired electron does not play an important role in the excited states analyzed in this work. In the calculations, the SOMO is found at −6.18 eV (corresponding to the 195A molecular orbital), which is ~1.7 eV deeper in energy than the “HOMO group”. The SUMO is found at −1.93 eV (the 201B molecular orbital), i.e., 0.7 eV above the “LUMO group”. We have modified Fig. S11 in the supplementary materials, to denote SOMO and SUMO in the molecular orbital energy diagram. Since the unpaired electron “comes from” the Cu metallic center, SOMO and SUMO wavefunctions can be qualitatively traced back to the *d* orbital of copper (see new Fig. S12), which spills over the naphthalocyanine arms. Therefore, as correctly pointed out by the reviewer, SOMO and SUMO do not participate in the two (degenerate) excited states ES3 and

ES4 of the monomer, which are the relevant ones to our work given their energy and optical activity, as they involve orbitals in the HOMO and LUMO groups. The main effect of the SOMO orbital is to shift the energies of the “A-(spin up)“ orbitals, so that, in practice the ES3 and ES4 look very much like excited states of singlet molecules. The SUMO is indeed involved in the ES5 excited state, which is a dark state.

As for the dimer, we have optimized the geometry with both triplet and singlet multiplicities under unrestricted conditions. We found that the triplet configuration leads to a lower total energy. This fact was further verified by running a single point calculation with triplet multiplicity on the singlet geometry, which again gave a lower total energy. All these pointed to the fact that the true ground state of the dimer has a triplet multiplicity. As a further test, prompted by the reviewer’s comment, we have now performed a singlet TDDFT calculation on top of the singlet geometry, even though this is potentially inappropriate, as the singlet state would provide an incorrect reference state for the calculation. As expected, we indeed find a negative first excitation energy, with a triplet state. Regarding the molecular orbitals of the dimer, we find two SOMO-derived molecular orbitals in the A-list, at -6.21 eV, which is approximately 1.7 eV lower than the HOMO group, and two SUMO derived molecular orbitals in the B-list, at -1.97 eV, which is 0.7 eV higher than the LUMO group. As can be seen in the new Fig. S15 of the revised supplementary materials, similarly to the monomer case, the corresponding molecular orbitals basically reflect the *d* orbitals of the metallic centers.

We agree with the referee that a more focused characterization of the spin multiplicity of the molecules enhances the value of our work, so we have modified both the main-text and the supplementary materials in several parts accordingly (marked as **REV#1**).

I do not understand why the authors operate with the transition dipole (Eq.3) and then define the "space resolved version of the transition dipole matrix element" (below Eq.3) as mu_alpha(x,y,z)=psi^*_i alpha psi_j, with alpha being x, y, or z. The space resolved version of the transition dipole is the transition density (or transition current density, requiring different treatment), which can be straightforwardly obtained from quantum-chemical software. Transition density can be readily used for the evaluation of plasmon-exciton coupling using the model proposed in the SI by evaluating the quasi-static potential phi instead of the electric field in Supp. Eq.(19-21). The coupling is then obtained as an integral over transition density multiplied by phi (that squared, to obtain the "oscillator strength"), rather than by Eq.(18). In fact, if I am not mistaken, the present theory, Eq.(18), is incorrect as it implies that the quasi-static potential of the plasmon can be obtained from the electric field as phi=x*Ex+y*Ey+z*Ez, which would be incorrect. I suggest that the authors use the well-accepted and easy-to-use theory employing the transition density. Alternatively, the authors can evaluate the transition current density and apply the appropriate theory. Correcting this point is critical, in my opinion.

Related to the comment above, I strongly suggest that in the Supp.Fig. S7,S8 and Fig.4 of the main text the transition density is shown. It is much more physically relevant and representative than the present plots as the transition density reveals the microscopic charge distribution (and hence dipoles). I discourage the authors from using the concept of the "differential form of the

transition matrix element" as it is a poorly defined quantity depending on the choice of coordinates and brings limited insight.

We thank the referee for raising these points. We agree with the referee that the transition density is a well established quantity and is useful to understand, from a microscopic perspective, why a given excited state is (or is not) optically active. In preparing our work we decided to go one step further and present what we called “space resolved transition dipole matrix elements”. This quantity has the advantage of being one integral away from the usual transition dipole matrix elements, thus being, in our opinion, more directly interpretable. To make a practical example, a dark (or optically active) state can be readily recognized acknowledging the parity of the components in the “space resolved transition dipole matrix element”. Doing so with the transition density is still possible, but it is intuitively more difficult, as one has to do the abstraction work of multiplying the shown function by x, y or z, before performing the integration. In any case, stimulated by the reviewer’s comment, we have dug more deeply and found out that the quantity we used is also called “transition dipole density” (within the Multiwfn tool) or “transition dipole moment density” (see for instance Zeyu Liu, Tian Lu, Qinxue Chen, Carbon 165 (2020) 461e467), even though it is less known than the transition density.

In preparing our original manuscript, we refrained from also showing the transition density, for brevity. We have realized that our presentation, and the motivation behind it, could not be as clear as we thought. For the sake of clarity and completeness, in the revised version we now provide both the transition densities (in the supplementary materials) and the transition dipole densities, and we have rewritten several parts in the manuscript and in the supplementary materials accordingly. We have decided to keep the plots of the “space resolved transition dipole matrix elements”, employing the more standard term “transition dipole density”, and clearly laying down the visual advantages that are obtained from using it. Nevertheless, we also show and comment on the transition densities of the analyzed excited states (please, have a look at the new figures S16 and S17 in the revised supplementary materials).

As for the modelling of the electric field in the STM cavity, we are very thankful to the referee for raising this point; we have realized that the approach used to model the electric field in the cavity, inspired by previous works in the literature, was not entirely correct. We had assumed that the quasi-static potential of the plasmon can be obtained from the electric field as phi=x*Ex+y*Ey+z*Ez. Since our concept never involved accurate modelling the electric field of the cavity, we have removed these results and have taken into account the effect of the STM tip by integrating the transition dipole density in a restricted region (~ 1 nm), in accordance to the spatial localization enabled by the nanotip of the STM. This corresponds to considering the electric field only in the region close to the nanotip and zero elsewhere. Although this is the most rudimental way of describing the electric field enhancement underneath the nanotip, it does not suffer from the problem mentioned by the referee. As shown in the revised manuscript, this simple model still provides qualitative agreement with the experimental results.

All these changes are now included in the new redaction of the main text and supplementary materials and marked by the label **REV#2** in the main-text and supplementary materials.

In Fig. 2a the authors show the photocurrent that is slowly changing as bias voltage is applied. This does not seem like an abrupt change of the photocurrent when a threshold is passed, as suggested in Fig.2c,d. How do the authors explain this behavior? What mechanism is behind it? Does it have any implications for the interpretation? How does it relate to the previous photocurrent studies in STM on molecules that suggested that substrate reorganization stands behind gradual onsets?

The reviewer correctly points out that the I-V curve recorded in presence of the laser pulses (Fig. 1e, main-text) shows that the polarity of the photocurrent gradually switches from positive to negative at ~ −0.3 V, rather than an abrupt change. This behavior has also been observed in previous photocurrent studies using CW lasers. (*Nature* **603**, 829–834 (2022); *Nano Letters* **25** (1), 578-585 (2024); *Nature Communications* **16** (1), 6039 (2025)). The polarity change can be explained by the relative alignment of the Fermi-level of the substrate with the electronic levels of the molecule and the competition between different photocurrent channels at different biases. Such a mechanism has been discussed in detail in these earlier works.

In Fig.3 the comparison of the FFT of the autocorrelation function alongside with the electroluminescence spectrum. The latter is evidently much broader. How do the authors understand that? This is related to my previous question about comparing the FFT of the autocorrelation to the absorption spectrum.

The broader lineshape of the electroluminescence spectrum compared to the FFT spectrum stems from the fundamentally different excitation mechanisms. The electroluminescence is driven by the electron tunneling, which can populate a broad manifold of vibronic levels in the excited state. Multiple radiative transitions, such as from the nth vibrational level of the excited state to the mth vibrational level of the ground state (n-m transition), will lead to broad spectral bandwidth in the electroluminescence spectrum. In contrast, in the photocurrent work, the excitonic wavepacket is launched from the 0th vibrational state of the ground electronic state. The time-resolved photocurrent signal primarily originated from the 0-0 transition, which leads to a much cleaner and narrower FFT spectrum. The origin of the broader spectral bandwidth of the electroluminescence spectrum in contrast to the absorption spectrum has been previously discussed, for example in the work by Imada et al. *Science* **373**, 95-98 (2021).

Third paragraph of the section "Atomic-scale control of dark and bright triplet excitons": I do not understand why the observation of four spectral peaks for the coupled dimer implies site-dependent excitonic coupling (exciton-exciton coupling). It rather implies the well-known breaking of selection rules and the possibility to access the dark excitons as well. The authors should show full spectrally filtered (electroluminescence or photoluminescence...) photon maps at the energies of the observed peaks to reveal their origin. I also want to point out that the possibility to measure dark states has been well known in the community for almost a decade (as correctly cited by the authors).

We agree that the observation of four peaks in the coupled dimer is a consequence of the intermolecular coupling and the breaking of selection rules. Although measuring dark states using

STML has been demonstrated earlier, the dark states at higher energies usually exhibit much less intensity compared with the lower-energy states.

We note that acquiring full spectrally filtered photon maps at each peak energy are experimentally demanding due to the weak STML yield of CuNc and the long associated acquisition times. Nevertheless, a comparison of the FFT spectra retrieved from the time-resolved traces with the STML spectra measured at identical positions of the nanotip over the molecular dimer is shown in Fig. R1. The bright E1 (1671 meV) state is apparent also in the STML measurements at the edges of the dimer, whereas, the dark E4 (1698 meV) state is barely observable in STML at the center of the dimer. This is likely due to very short decoherence time of the dark states, making them hard to detect in emission-based spectroscopy.

We have now included the STML spectra measured on the molecular dimer as Fig. S9 in the revised manuscript; please see **REV#3** in the main-text and supplementary materials on page 8.

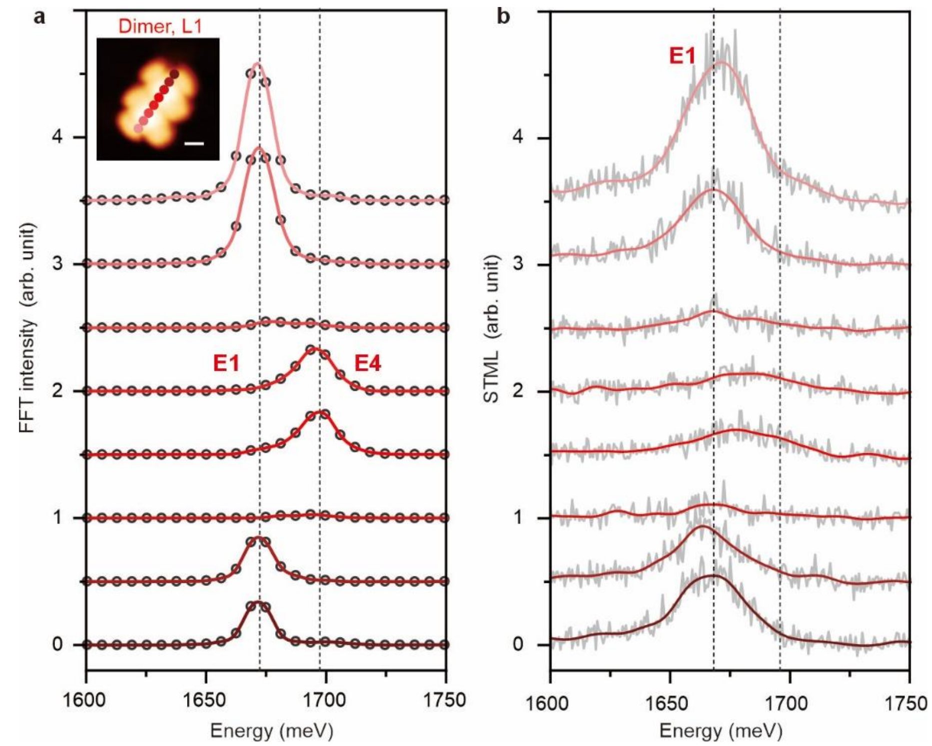

REV#3 Fig. R1 | Comparison of spectra obtained from FFT of the time-resolved traces and STML. a, A series of FFT spectra retrieved from time-resolved photocurrent measurements at eight equidistant positions of the nanotip over the CuNc dimer (red dots in the inset of **a**), adopted from Fig. 5a of the main-text. **b,** STML spectra measured at the identical positions of the nanotip over the dimer as in **a** at a bias of −2.2 V and tunneling current of 50 pA. An integration time of 30 seconds was used for each spectrum. The spectra in the STML measurements only show the bright excitonic state (E1, 1671 meV), whereas the dark excitonic state (E4, 1698 meV) was not observed at the center of the molecular dimer. This is likely due to very short decoherence time of the dark states, making them hard to detect in emission based spectroscopy. The spectra have been vertically shifted in **a** and **b** for clarity.

I find the conclusions very generic and vague. What is th actual concrete finding of this study that is unique to it? How concretely is it relevant to light-harvesting complexes or singlet fission?

We thank the reviewer for this comment. We have demonstrated an experiment to track the coherent evolution of an excitonic wavepacket in a single molecule, in real-space and real-time. We directly measured the intrinsic coherence time of an exciton (~70 fs) in an isolated molecule, and quantitatively showed that intermolecular coupling reduces the excitonic coherence time. Dynamics unfolding in the excited state of molecules is relevant to many physical processes such as electronic excitation energy transfer, conical intersections, and singlet fission. While we did not study light-harvesting complexes or singlet fission directly in this work, our ability to track the exciton coherence in single and coupled molecules provides the essential methodology required to explore these possibilities in future works.

There is a typo in Supp. Eq.10

Thank you for pointing this out, we have now rectified it.

Reviewer #2 (Remarks to the Author):

In this manuscript entitled "Orbital-resolved imaging of coherent femtosecond exciton dynamics in coupled molecules", Luo et al. report an impressive implementation of femtosecond wave-packet interferometry in an STM junction, enabling orbital-resolved mapping of excitonic decoherence at the single-molecule level. The authors further compare monomer and dimer cases to reveal the influence of intermolecular coupling on the coherence time. The modeling for the molecular transition dipoles and the tip-enhanced electric field is also qualitatively consistent with the experimental observations. The technical execution is outstanding, and the work represents a notable advance in ultrafast STM spectroscopy. I fully support its publication in Nature Communications, with only a few items that the author may consider addressing that might help the readers to better appreaciate this work.

We would like to sincerely thank the reviewer for their kind words and for judging our study as "an impressive implementation of femtosecond wavepacket interferometry in an STM junction", and "notable advance in ultrafast STM spectroscopy". We are glad to make all possible efforts to increase the clarity of all the points raised and to meet the expectations of the reviewer.

The manuscript presents the measured coherence times for the monomer and dimer but does not sufficiently discuss the microscopic origin of the decoherence in the single-molecule limit. Is it mainly driven by electronic dephasing, vibronic coupling, substrate-induced fluctuations, or hybridization with the metallic environment? Since the authors show that reducing the NaCl thickness changes the coherence time, it would be helpful to clarify the relative roles of molecule–substrate coupling and to comment on possible strategies to tune T_2 using different insulating layers or substrates. It would also be useful to compare the observed values with known dephasing rates in related molecular systems.

We thank the reviewer for this insightful comment. The decoherence time of the excitons in CuNc molecules on a 4 ML thick NaCl is ~ 70 fs (please see Fig. R2 below), which is very similar to that of a molecule measured on a 3 ML thick NaCl film. They are certainly slower than the case of a molecule on a 2 ML thick NaCl film (~ 50 fs). For comparison, the decoherence time of a Zinc Phthalocyanine (ZnPc) molecule estimated from spectral linewidth in similar experimental conditions as in the current work will be ~ 300 fs (*Nature Nanotechnology* **17**, 729–736 (2022)).

The non-varying behavior of the decoherence time on higher thicknesses of the insulating film and the estimated longer coherence times of the Phthalocyanine molecules imply that the observed behavior is an intrinsic property of the molecule. It is also noteworthy that the intensity of the electroluminescence of CuNc is significantly weaker than many other molecules and in an earlier work could not even be measured (*Nature Nanotechnology* **15**, 105–110 (2020)), which further connotes to the faster decoherence rates in the excited state of CuNc. The origin of decoherence in the excited state of the molecule is primarily vibronic coupling, which could arise from a conical intersection. However, we must emphasize that a detailed study would be required to attest this conjecture.

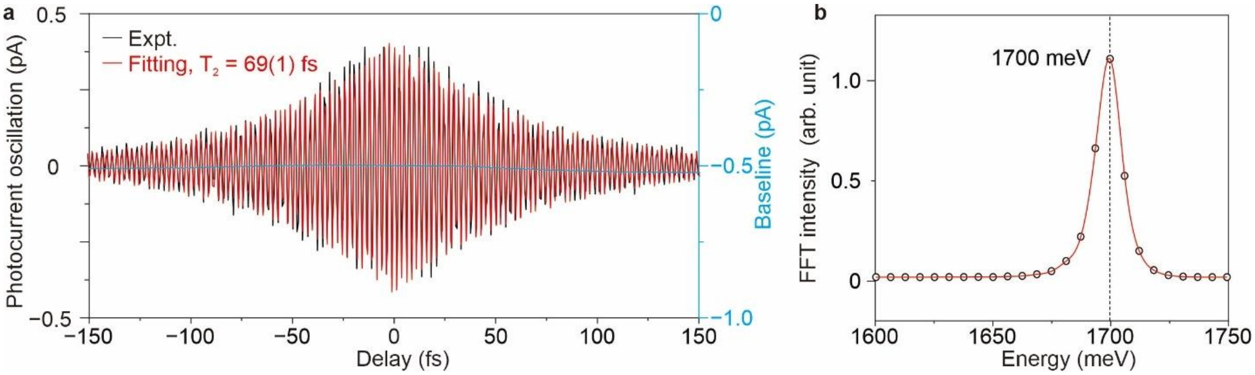

REV#4 Fig. R2 | Quantum decoherence of excitons in a single CuNc molecule on a 4ML thick NaCl film on Ag(111). a, Time-resolved photocurrent measured at the lobe position of a single CuNc molecule present on top of a 4ML thick NaCl film. The nanotip was stabilized at 1 V and 2 pA on the molecular lobe, then the bias was changed to −1 V and the nanotip was further approached by 80 pm before starting the time-resolved measurements. The oscillatory component (black curve) of the photocurrent was extracted by subtracting a smoothed baseline (blue curve) from the raw time trace. **b,** Fast Fourier transformation (FFT) spectrum of the time trace in **a**, indicating the position of the excitonic peak in the monomer. The excitonic peak in the case of a molecule on 4 ML is slightly blue shifted to ~ 1700 meV compared to the peak position in 2 ML (~ 1679 meV) and 3 ML thick NaCl (~ 1687 meV), likely due to lower electromagnetic screening of the molecule from the underlying Ag(111) substrate.

We have now included the above figure as Fig. S7 in the revised manuscript. Please see **REV#4** (new Fig. S7) in supplementary materials on page 6 and main-text.

Prior STM studies have shown that the local tip–sample electric field can shift molecular orbital energies and thereby modify excitonic transitions. In Fig. 5c, the slight peak shifts with lateral tip position may reflect such effects. The authors should clarify whether systematic tip-height or lateral-position dependence was observed, and whether the extracted exciton energy and T_2 remain consistent under such perturbations. It would also be useful to comment on whether adjusting the tunneling channel via bias affects the measured coherence. A quantitative analysis of peak energy versus tip position would strengthen the interpretation.

Figure R3 shows the time-resolved photocurrent traces (black curves) of the measurement corresponding to the FFT spectra shown in Fig. 5c (main-text) along with the two-level fitting of the traces (red-curve). The time-resolved traces from **a** to **f** are in one-to-one correspondence to the FFT spectra measured from the top to the bottom positions of the nanotip in the monomer (Fig. 5c, main-text). Both the decoherence time and spectral position of the excitons are virtually insensitive to the lateral position of the nanotip over the monomer. We have now included the Fig. R3 in the revised manuscript as Fig. S10, please see **REV#5** in main-text and supplementary materials on page 9.

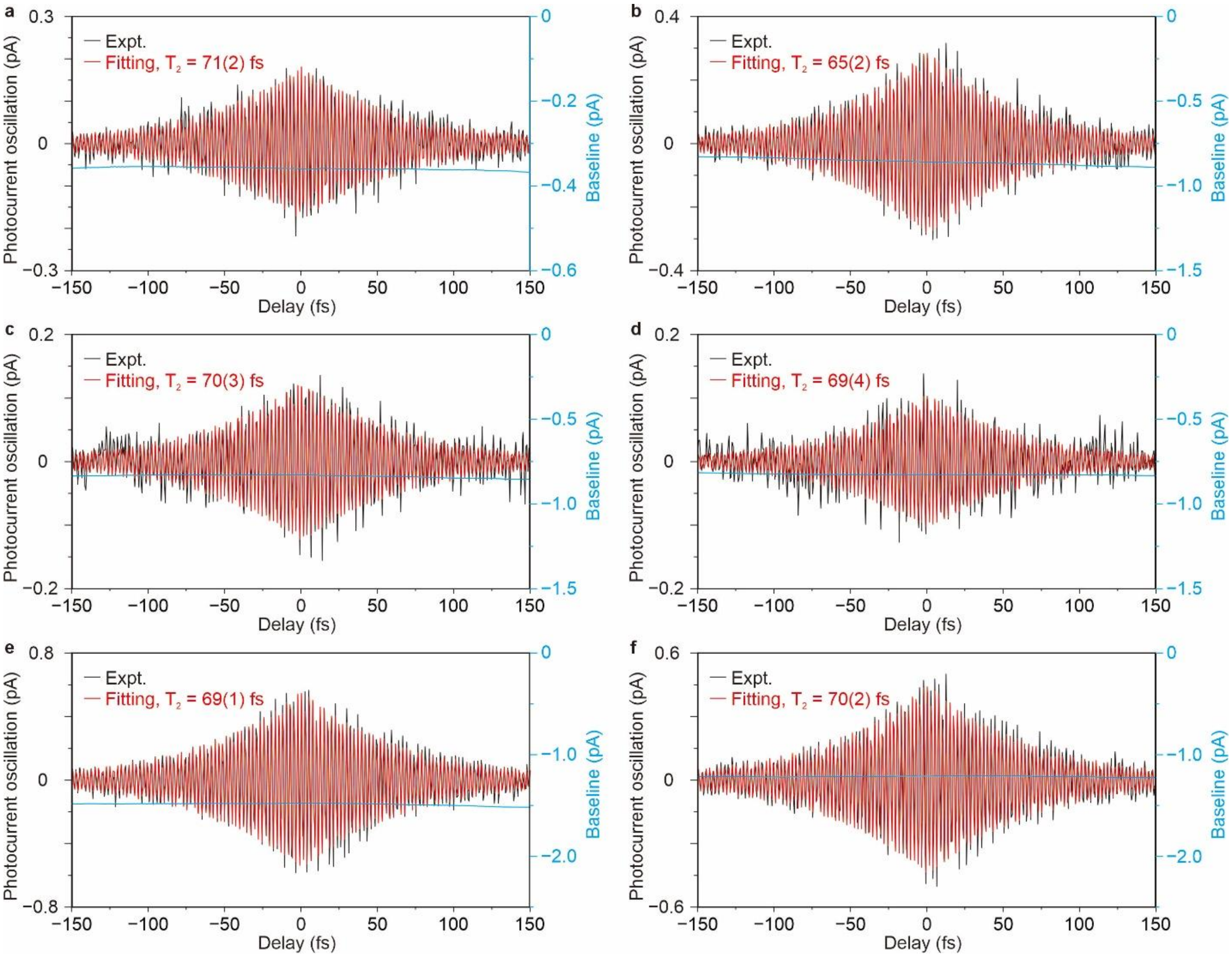

REV#5 **Fig. R3 | a-f:** Time-resolved photocurrent traces measured as a function of the delay between pulse-1 and pulse-2 at six equidistant positions of the nanotip over the CuNc monomer (Fig. 5c, main-text). The oscillatory component (black curve) of the photocurrent was extracted by subtracting a smoothed baseline (blue curve) from the raw time trace. The FFT spectra of the time-resolved traces are shown in Fig. 5c (main-text). Measurement in panels **a** to **f** correspond one-to-one to the FFT spectra from top to bottom positions of the nanotip over the molecule. The decoherence time as well as the spectral position of the excitons is virtually insensitive to the lateral position of the nanotip over the molecules.

Regarding the variation in the tip-height; owing to the exponential dependence of the photocurrent on the size of the nanocavity, on a mere change in the tip height of < 100 pm, the photocurrent vanishes (Fig. 1g, main-text). This strong sensitivity prohibits a systematic investigation into the variation of the decoherence time on the size of the nanocavity.

Figure R4 below shows the comparison of the time-resolved photocurrent traces recorded at the biases of 0 V and -1 V in the STM junction. Spectral position of the exciton is invariant to the

applied bias in the STM junction. Nonetheless, there is a minor variation of the decoherence time on the applied bias, suggesting its possible dependence on the tunneling channel. Ascertaining the underlying mechanism behind this dependence requires a more detailed investigation, which is out of the scope of the current work and plausibly requires using a molecule with significantly longer decoherence times for a more sensitive observation of variation of such parameters in the experiments. We hope for the understanding of the reviewer.

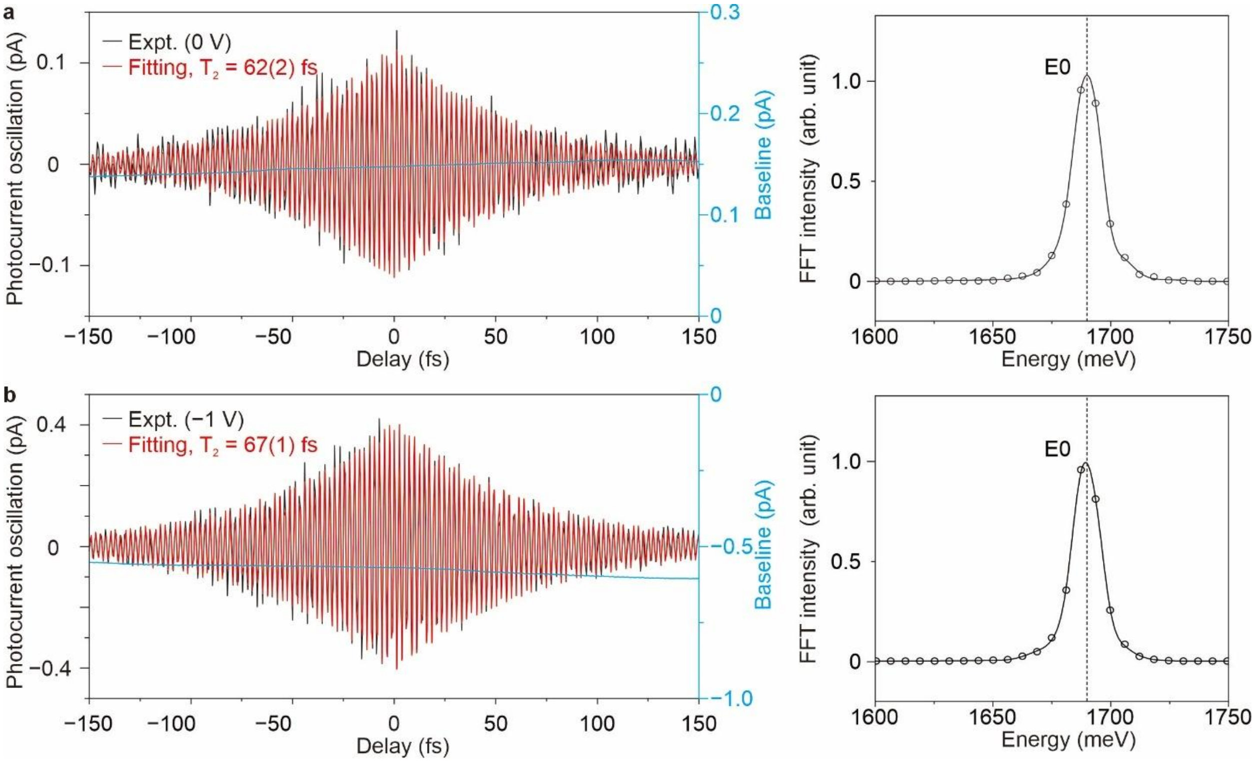

Fig. R4 | Time-resolved photocurrent traces recorded at the biases of 0 V (a) and −1 V (b) in the STM junction. The nanotip was stabilized at 1 V and 2 pA on the molecular lobe of CuNc molecule on a 3ML NaCl film, then the bias was changed to 0 V (a) and −1 V (b) and the nanotip was further approached by 160 pm before starting the time-resolved measurements. The oscillatory component (black curves) of the photocurrent were extracted by subtracting a smoothed baseline (blue curves) from the raw time traces. The molecular spectra obtained by fast Fourier transformation of the time traces show identical peak positions (right panels).

In Fig. S2b, a small shoulder appears on the low-energy side of the FFT spectrum with an amplitude of ~0.1, which is comparable to the E_4 peak of the dimer in Fig. 4b. The origin of this feature is unclear. The authors should comment on whether it arises from vibronic replicas, instrumental artifacts, or weak excited states, and whether it is reproducible over multiple measurements and molecules. Some clarification or control data are needed to rule out artifacts.

The experimental time-traces presented in this work have been measured for different molecules with different shapes of the nanotip over different days (and months). Despite these variations, the retrieved decoherence times and the spectral positions of the excitons were consistently reproducible.

The amplitude of the photocurrent for a CuNc molecule deposited on a 2 ML thick NaCl insulating film is weaker compared to that of a molecule present on 3 or 4 ML thick NaCl films. This weak signal produced the artefact at the low-energy side of the excitonic peak, which was not observed in other measurements across different molecules or on thicker (3 – 4 ML) NaCl films. We therefore attribute this feature to the noise floor or a measurement artifact.

The dimer results show both energy splitting and a reduced coherence time. Have the authors investigated dimers with controlled intermolecular separations or different geometries? Such data would be valuable for understanding how coupling strength influences exciton delocalization and decoherence, and whether the bright/dark nature evolves gradually as the coupling is tuned. It would also be interesting to know whether charge-transfer excitonic character appears at smaller separations.

We have investigated CuNc oligomers with varying intermolecular separations and relative orientations. Figure R5 below shows measurements performed at different intermolecular separations between the CuNc molecules than the one shown in the main-text. For larger separations, neither the decoherence time nor the spectral position of the exciton is affected compared to a monomer (red and black curves in Fig. R5d). Specifically, at large separations of ~1.8 nm and ~3.0 nm, (see Fig. R5a and R5b), the exciton decoherence time and energy remain almost identical to the monomer case, indicating electronically uncoupled behavior.

For a CuNc dimer with nonparallel alignment of the molecular axes (inset of Fig. R5c), there is negligible shift of the bright E1 excitonic state compared to a dimer with parallel alignment of the molecular axes (Fig. 5a, main-text), as shown in Fig. R5c and Fig. R5d. Furthermore, their coherence times are also similar in these two configurations.

Charge-transfer excitons might exist at smaller intermolecular separations between the CuNc molecules in the dimer as the reviewer correctly points out. However, their presence in the current experiment could not be confirmed. A heteromer would be more suited to investigate such charge-transfer excitons as it will (may) lead to formation of distinct (new) states in the spectra, which could not be explained by only accounting for intermolecular interactions, as in the current work.

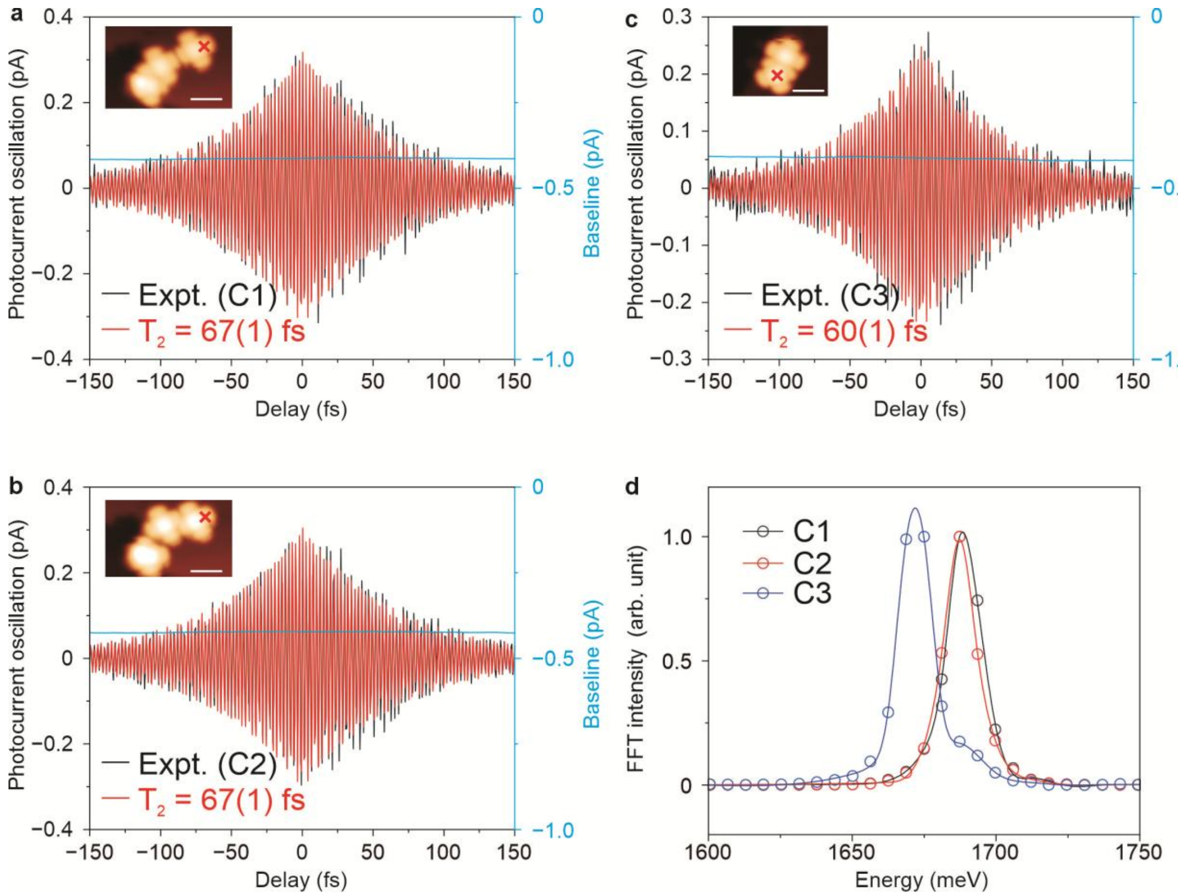

Fig. R5 | a, b, Time-resolved photocurrent measurements recorded for CuNc molecules with larger intermolecular separations; ~ 3 nm (**a**) and ~ 1.8 nm (**b**), compared to the smaller separation shown in the main-text (~ 1.6 nm, Fig. 5). The decoherence times of the excitons retrieved by a two-level fitting is similar to the case of a monomer (~ 70 fs), also, the spectral position of the excitons is not affected (black and red curves in **d**). **c**, Time-resolved photocurrent measurement for a dimer with nonparallel alignment of the molecular axes (inset). The intermolecular separation between the molecules is ~ 1.6 nm. The oscillatory component (black curve) of the photocurrent was extracted by subtracting a smoothed baseline (blue curve) from the raw time trace. **d,** Comparison of the FFT spectra obtained from the measurements shown in **a-c**. The legend corresponds to the dimer configuration as indicated in **a-c**. The spectral position of the bright E1 exciton (blue-curve in **d**) and its decoherence time (~ 60 fs as shown in **c**) are similar to the case of the dimer with parallel alignment of the molecular axes (Fig. 5, main-text). In the measurements, the nanotip was stabilized at 1 V and 2 pA on the molecules (red crosses in the inset), then the bias was changed to −1 V and the nanotip was further approached by 160 pm before starting the time-resolved measurements. Exciton decoherence times (T_2) were determined by fitting the time-resolved photocurrent traces. Scale bars in the inset correspond to 2 nm.

The excitation bandwidth (1450–1900 meV) is broad enough to drive vibronic transitions and create multi-mode wave packets. How does this affect the extracted coherence times? Are

vibrational coherences contributing to the dephasing, and could some of the FFT features originate from vibronic beating? A brief discussion or theoretical estimate separating electronic and vibronic contributions would help clarify this point.

An electronic wavepacket launched from the ground state to the excited state can have contributions from multiple vibrational levels, as the reviewer correctly points out. This superposition of the states is determined primarily by the bandwidth of the exciting laser pulses and the Frack-Condon factors. Fingerprints of different vibrational levels in the electronic wavepacket were also observed in the current experiments, nonetheless, their contributions in the wavepacket were rather low.

Figure R6 below shows a comparison of the FFT spectrum obtained from the time-resolved photocurrent measurements of a CuNc monomer with the STML spectrum in the logarithmic scale. Weak vibronic peaks can be seen around ~ 1760 meV (~ 650 cm^{-1} in the STML spectrum). Since the observed contribution of the vibronic levels in the electronic wavepacket is rather low, it prohibits a quantitative study on its influence on the dephasing dynamics in the current work. Molecular systems with longer decoherence times will hopefully enable a better understanding towards this, and will be the focus of a future work.

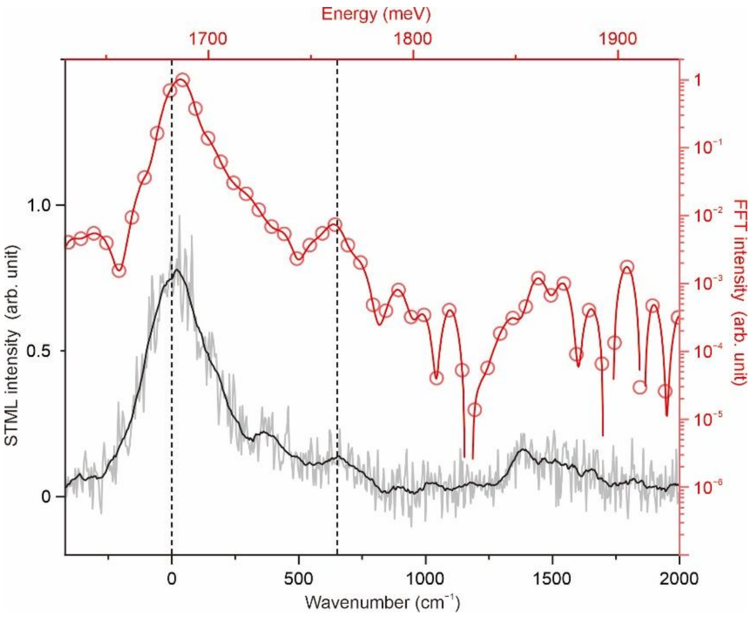

Fig. R6 | Comparison of the molecular spectra obtained by STML (black curve, left axis) and by fast Fourier transformation of the time-resolved photocurrent trace (red curve, right axis) measured on a CuNc monomer. The STML spectrum was measured on the CuNc lobe at a bias of −2.5 V and a tunneling current of 50 pA. The FFT spectra is plotted on the logarithmic scale, which shows very weak vibronic peaks, at around ~ 1760 meV. The spectra are displayed as a function of energy shifts (bottom axis) with respect to main excitonic emission peak (~ 1687 meV).

Lastly, just a quick note: as a fellow STM researcher, I cannot agree more that phthalocyanine derivatives are a favorable system for proof-of-principle studies, particularly when demonstrating technical advancements such as those presented in this work. However, this is also a decades-old model system that has been extensively investigated from many perspectives. I would look forward to seeing more insights drawn from other molecular systems, which I expect could provide even more valuable and exciting chemical and physical understanding.

We would like to thank the reviewer for this comment. We fully agree with the reviewer that Phthalocyanine derivatives are a well-studied model system in STM studies, owing to the relative ease in handing them in the experiments. We have benchmarked wavepacket interferometry to track exciton dynamics in single and coupled molecules in the current work and hopefully, we will be able to move to more complex systems in the future, where we can get deeper physical insights into ultrafast processes at the single molecule level.

Reviewer #3 (Remarks to the Author):

I co-reviewed this manuscript with one of the reviewers who provided the listed reports. This is part of the Nature Communications initiative to facilitate training in peer review and to provide appropriate recognition for Early Career Researchers who co-review manuscripts.

We would like to sincerely thank the reviewer for their time and efforts to work on our manuscript.

Reviewer #4 (Remarks to the Author):

In this manuscript, the authors demonstrate real-space observation of coherent exciton dynamics using ultrafast STM. Specifically, a CuNc molecule adsorbed on an ultrathin NaCl layer within a plasmonic STM junction is excited by 15-fs visible laser pulses, and the resulting photon-induced tunneling current is measured with high precision to visualize the ultrafast exciton dynamics at the single-molecule level. Previous studies in similar systems have reported excitonic resonances and energy transfer under continuous-wave illumination, and more recently, ultrafast dynamics have been probed using THz-STM. However, to the best of my knowledge, this work represents the first demonstration of exciton dynamics in a single molecule driven and detected using few-cycle visible/near-infrared pulses. While ultrafast STM has previously been applied to coherent phonons in oxide thin films, it has not yet been extended to molecular excitons. Therefore, this study constitutes a significant and timely advancement toward ultrafast molecular spectroscopy using visible/near-infrared STM.

Ultrafast STM techniques that enable combined atomic-scale spatial resolution and femtosecond temporal resolution are rapidly advancing and are attracting growing attention due to their potential impact in quantum science and nanoscale condensed-matter research. The methodology demonstrated here is at the forefront of the field, the experimental data are of excellent quality, and the analysis, including first-principles calculations, is rigorous and thorough. The discussion of prior work is appropriate and the cited literature is relevant. Based on these considerations, I believe this manuscript will attract broad interest within the nanoscience and nanotechnology community, and its scientific merit supports publication in Nature Communications.

We sincerely thank the reviewer for their kind words and for recognizing a series of novel elements in our work, which are expected to have “impact in quantum science and nanoscale condensed-matter research”. We have made all possible efforts to improve the clarity of the presentation in order to address all the points raised and to meet the expectations of the reviewer.

Minor comments:

1. The observed excitonic response may depend on the tip–molecule distance, for example through tip-induced Stark shifts, tip-induced orbital hybridization, or modifications to the exciton dephasing time arising from tip–molecule interactions. Although this does not affect the main conclusions, a brief discussion on this point would strengthen the manuscript.

The decoherence time of the excitons and their spectral positions are insensitive to the lateral position of the nanotip over the CuNc molecule. Figure R3 below shows the time-resolved photocurrent traces (black-curve) corresponding to the FFT spectra shown in Fig. 5c (main-text) along with the two-level fitting of the traces (red-curve). The time-resolved traces from **a** to **f** are in one-to-one correspondence to the FFT spectra measured from the top to the bottom positions of the nanotip in the monomer (Fig. 5c, main-text). Both the decoherence time and the spectral

position of the excitons are virtually insensitive to the lateral position of the nanotip over the monomer. We have now included the Fig. R3 in the revised manuscript, please see **REV#5** in main-text and supplementary materials on page 8. Please also see our response to reviewer 2.

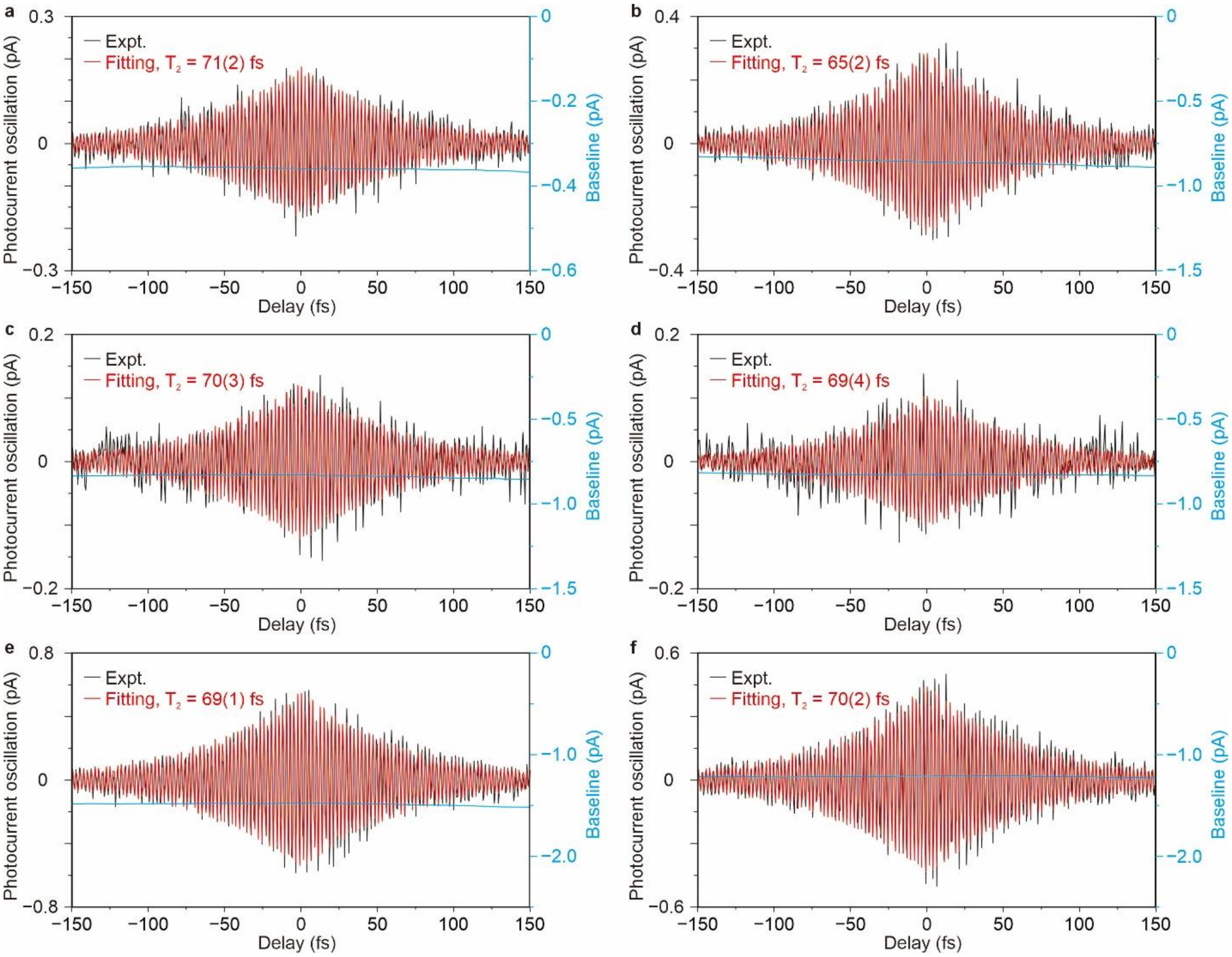

REV#5 **Fig. R3 | a-f:** Time-resolved photocurrent traces measured as a function of the delay between pulse-1 and pulse-2 at six equidistant positions of the nanotip over the CuNc monomer (Fig. 5c, main-text). The oscillatory component (black curve) of the photocurrent was extracted by subtracting a smoothed baseline (blue curve) from the raw time trace. The FFT spectra of the time-resolved traces are shown in Fig. 5c (main-text). Measurement in panels **a** to **f** correspond one-to-one to the FFT spectra from top to bottom positons of the nanotip over the molecule. The decoherence time as well as the spectral position of the excitons is virtually insensitive to the lateral position of the nanotip over the molecules.

Figure R4 below shows the comparison of the time-resolved photocurrent traces recorded at the biases of 0 V and -1 V in the STM junction. Spectral position of the exciton is invariant to the applied bias in the STM junction, suggesting negligible Stark shifts induced from the applied bias

in the junction. Nonetheless, there is a slight variation in the decoherence time on the applied bias, suggesting its dependence on the tunneling channel. Ascertaining the underlying mechanism behind this dependence requires a detailed investigation, which is out of the scope of the current work and plausibly requires using a molecule with significantly longer decoherence times (e.g. ZnPc or PtPc). We hope for the understanding of the reviewer.

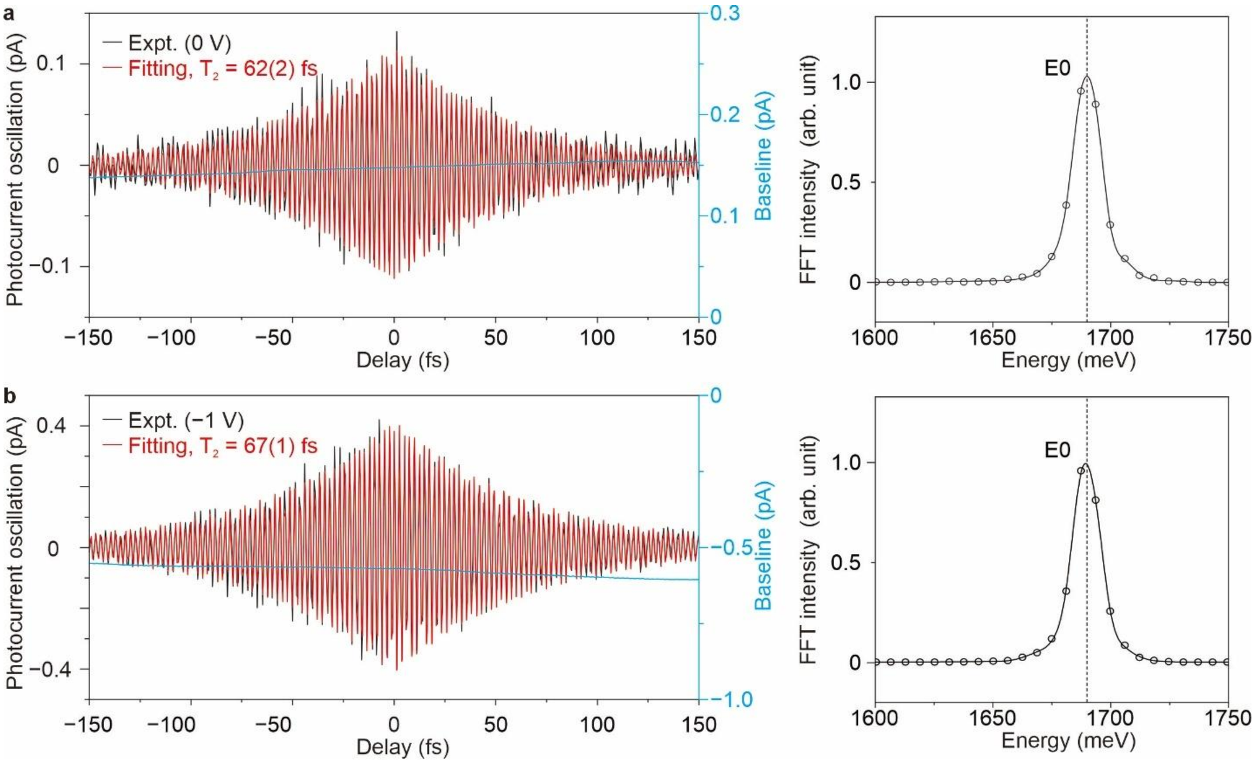

Fig. R4 | Time-resolved photocurrent traces recorded at the biases of 0 V (a) and −1 V (b) in the STM junction. The nanotip was stabilized at 1 V and 2 pA on the molecular lobe of CuNc molecule on a 3ML NaCl film, then the bias was changed to 0 V (a) and −1 V (b) and the nanotip was further approached by 160 pm before starting the time-resolved measurements. The oscillatory component (black curves) of the photocurrent was extracted by subtracting a smoothed baseline (blue curves) from the raw time traces. The molecular spectra obtained by fast Fourier transformation of the time traces show identical peak positions (right panels).

Regarding the tip-molecule distance dependence, owing to the exponential dependence of the photocurrent on the size of the nanocavity, on a mere change of < 100 pm, the photocurrent vanishes (Fig. 1g, main-text). This prohibits a systematic investigation into the variation of the decoherence time of the excitons on the size of the nanocavity (tip-molecule distance).

Although no variation of the excitonic decoherence time have been observed to vary with the tip-molecule distance, photocurrent imaging is highly sensitive to the tip-molecule distance. Figure R7 below shows the photocurrent imaging at three tip-molecule distances at a bias voltage of 0

V. Before acquiring the images, the nanotip was stabilized at 1 V and 2 pA on the molecular lobe, and then approached by 80 pm (left-most panel), 120 pm (middle panel) and 160 pm (right-most panel) at 0 V. At the closest tip-molecule distances (−160 pm, right-most panel), the photocurrent image is visibly different from the imaging at larger tip-molecule separations (−80 pm, left-most panel). This observation could stem from the stronger tip-molecule hybridization at closer distances, moreover, the relative contributions of different photocurrent channels (Fig. 2c, main-text) will also evolve with tip-molecule distance. Allerbeck et al. *Nature Communications* **16**, 10806 (2025) (please see Fig. 4 in the paper) have recently observed a very similar effect on imaging of atomic defects in monolayer of 2D materials. The paper is now cited as Ref. 30 in the main-text. Deciphering the detailed mechanism behind this observation will be focus of a future study.

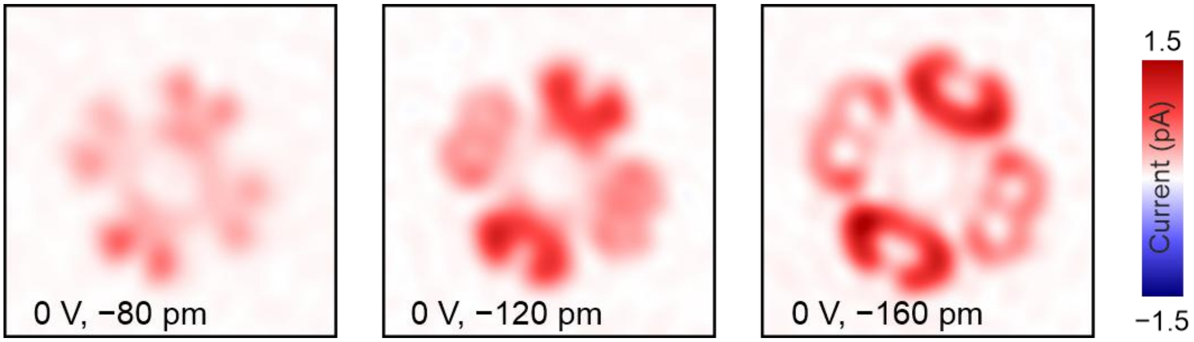

REV#6 **Fig. R7 |** Ultrafast photocurrent images (4.0 nm × 4.0 nm) of a single CuNc molecule on a 4ML thick NaCl film acquired with the open feedback loop (constant-height mode) at various heights of the nanotip as annotated in each individual panel. A laser power of 100 μW was used in the measurements. Before acquiring the images, the nanotip was stabilized at 1 V and 2 pA on the molecular lobe, and then approached by 80 pm (left-most panel), 120 pm (middle panel) and 160 pm (right-most panel) at 0 V. At closer tip-molecule distances (-160 pm, right-most panel), the photocurrent image is visibly different from the imaging at larger tip-molecule distance (-80 pm, left-most panel). This observation could stem from the stronger tip-molecule hybridization at closer distances (Ref. 30, main-text), moreover, the contributions of different photocurrent channels (Fig. 2c, main-text) will also evolve with tip-molecule distance.

We have now included the above figure as Fig. S3 in the revised manuscript, please see **REV#6** in main-text and the supplementary materials on page 3.

2. The strong sensitivity achieved in this work is likely supported by the localized surface plasmon resonance (LSPR) formed in the STM junction. At the same time, the enhanced optical near-field may also influence the excitonic response and photocurrent generation. While this does not alter the conclusions, I encourage the authors to include a brief discussion on the possible influence of LSPR, and, if feasible, present the STML spectrum to show the optical response of the plasmonic junction.

Spectral overlap between the molecular transitions and LSPR is critical to achieving a highly sensitive molecular response in the experiments, as the reviewer correctly points out. Figure R8 below shows a comparison of the STML spectrum of the plasmonic junction (LSPR) with the far-field spectrum of the laser pulses. We have now included this figure as Fig. S2 in the revised text; please see **REV#7** in supplementary materials on page 3.

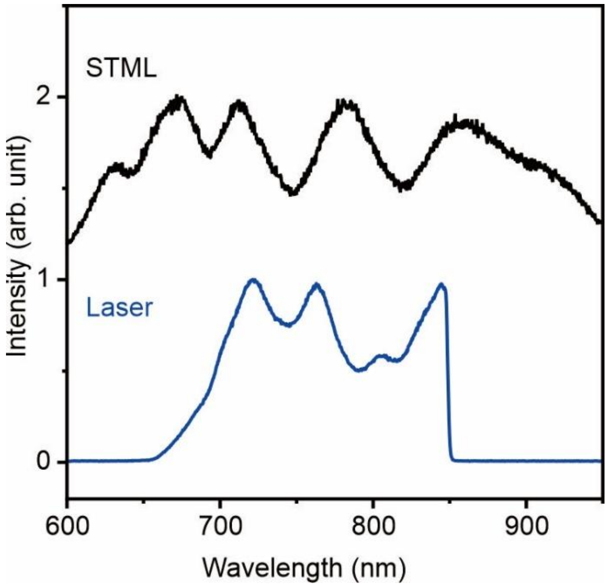

REV#7 Fig. R8 | Comparison of the STM induced electroluminescence (STML) spectrum of the plasmonic junction (black curve) with the spectrum of the incident laser pulses (blue curve). STML spectrum was measured on the bare Ag(111) surface at a bias of 2.5 V and tunneling current of 100 pA.

3. The plasmonic field enhancement and photocurrent sensitivity in STM-based optical measurements can significantly depend on the exact tip geometry and material condition. It would be helpful if the authors could comment on the reproducibility across multiple STM tips.

A strong plasmonic enhancement at the apex of the nanotips is required to detect photocurrents from the molecules. This necessitates extensive shaping of the nanotip apex by atomic indentation, and not all nanotips show efficiently strong plasmonic enhancement and thus detectable photocurrent signals. However, once a "bright" tip is obtained, the observed spectroscopic features are highly reproducible across different nanotips and on different days.

4. Given the intense electric field associated with the LSPR and 15 fs laser pulses, there may be concerns regarding laser-induced tip or molecule modification, heating, or photo-desorption effects. It would be helpful if the authors could clarify whether any potential contributions of these effects.

To demonstrate the absence of thermal effects in the measurements, we show below the time-frequency analysis of the time-resolved photocurrents measured on CuNc molecules and on bare NaCl surface. In case of bare NaCl (Fig. 3d, main-text), time-frequency analysis reveals broad

spectral distribution (Fig. R9a), which is localized in the temporal window of ~ 15 fs (Fig. R9b). However, in the case of time-resolved photocurrent measurements on a CuNc molecule, a single frequency component (Fig. R9c) corresponding to the excitonic peak can be seen in the broad temporal window extending up to ~ 200 fs (Fig. R9d). No frequency components corresponding to the carrier frequency of the incident laser pulses can be seen in the time-frequency plot, attesting to the absence of the thermal effects in the experiments. Presence of frequency components remnant of the carrier frequency of the laser pulses would have indicated thermal artefacts. At the pulse energies used in the current experiment, no photodesorption or tip-induced damage of the molecule was observed.

We have now included the Fig. R9 is the revised manuscript as Fig. S4; please see **REV#8** in supplementary materials on page 4.

It is worth mentioning that the pulse energy incident on the sample in the time-resolved measurements is ~ 600 fJ (~ 50 uW at 80 MHz), which is significantly low. Moreover, the laser-induced tunneling currents could be conveniently measured in the DC tunneling channel, thus, no additional lock-in measurements were used in the experiments, which avoids potential thermal artifacts that can arise from modulation-based measurements.

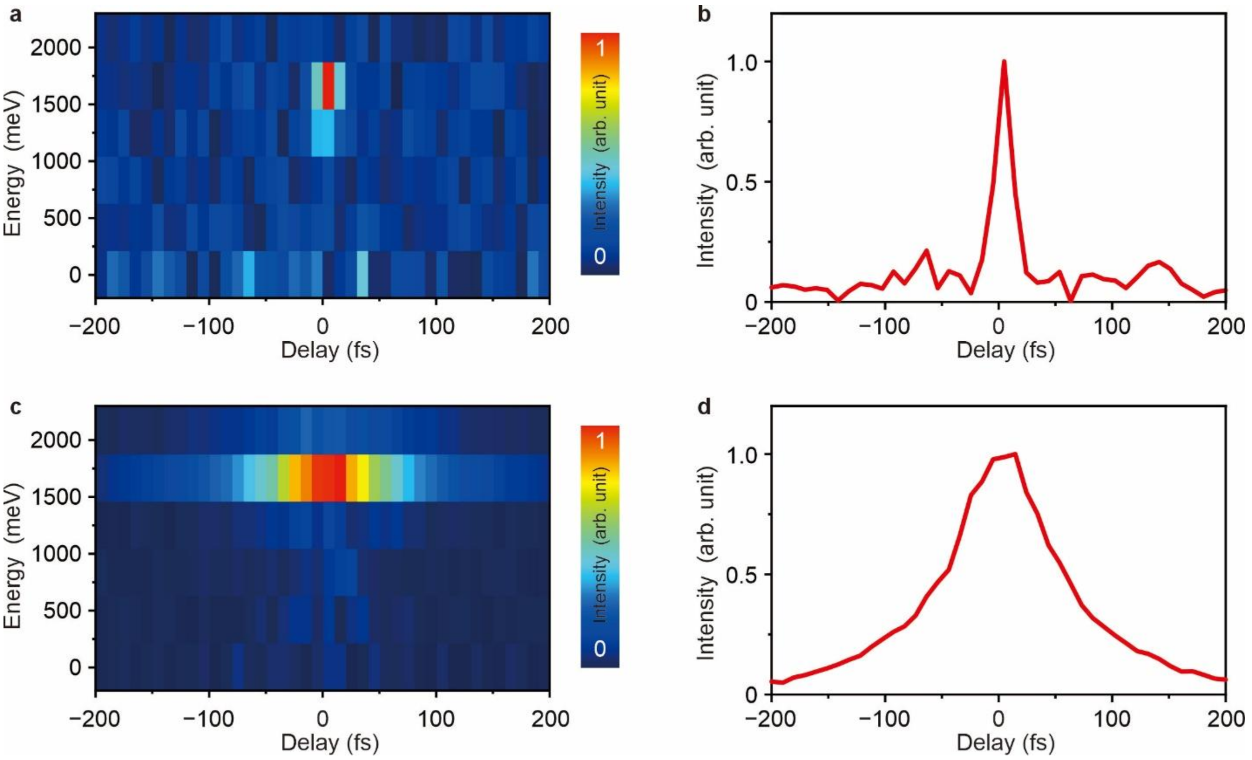

REV#8 Fig. R9 | Time-frequency analysis of the time-resolved photocurrent signal measured on bare NaCl (**a**) and on a CuNc (**c**) molecule, respectively**.** The analysis is performed on the time-domain signal shown in Fig. 3d (NaCl) and Fig. 3b (CuNc) of the main-text. **a, b,** Short-time Fourier transform spectrum was obtained using a 15-point (20 fs) Kaiser window with a shape parameter of 4. **b, d,** Temporal evolution of the intensity of the spectral envelope at ~ 1700 meV. In case of

bare NaCl (Fig. 3d, main-text), time-frequency analysis reveals a broad spectral distribution (Fig. R9a), which is localized in the temporal window of ~ 15 fs (Fig. R9b). However, in the case of time-resolved photocurrent measurements on the CuNc molecule, a single frequency component corresponding to the excitonic peak can be seen in the broad temporal window extending up to ~200 fs (Fig. R9c, R9d). No frequency component corresponding to the carrier frequency of the incident laser pulses can be seen in the time-frequency plot, attesting to the absence of the thermal effects in the experiments. Presence of frequency components remnant of the carrier frequency of the laser would have indicated thermal artefacts. At the pulse energies used in the current experiment, no photodesorption or tip-induced damage of the molecule was observed.

5. Since the photocurrent oscillations are used as a proxy to reconstruct exciton coherence, it may be useful to specify whether the pulse duration quoted (~15 fs) refers to the free-space pulse or the effective pulse duration in the plasmonic junction. If the latter is unknown, a short clarification acknowledging that plasmonic filtering may modify spectral phase would improve clarity.

We thank the reviewer for this comment. The pulse duration of the incident laser pulses is ~ 10 fs. The spectral shaping by the plasmonic response of the cavity narrows the incident spectrum, making the effective duration ~ 15 fs as characterized by the photocurrent measurements on bare NaCl. Figure R10 below shows the fringe-resolved autocorrelation (FRAC) trace of a ~ 10 fs long laser pulse. We have now included this FRAC trace as Fig. S5 in the revised manuscript, please see **REV#9** in the main-text and supplementary materials on page 5.

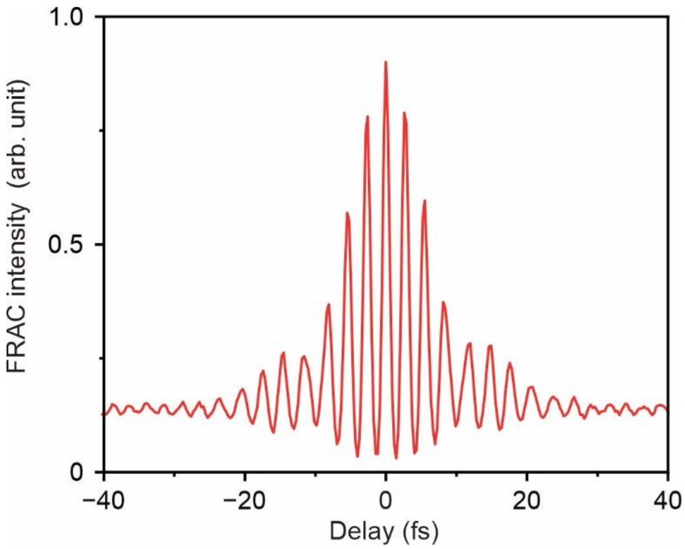

REV#9 Fig. R10 | Fringe-resolved autocorrelation trace of laser pulses (τ_{FWHM} ~ 10 fs) incident on the STM junction. A second harmonic generation based fringe-resolved autocorrelator (FRAC) with an ~ 20 μm thick BBO crystal was used to measure the duration of the pump and probe pulses with identical dispersion as in the optical path to the STM junction.

6. In some sentences, the term "exciton" appears to refer broadly to any excited-state transient wavepacket, whereas in others it refers to a spectroscopically defined bound state. I suggest ensuring consistent terminology or defining usage early in the manuscript.

We thank the reviewer for pointing out this inconsistency. To avoid ambiguity, we have carefully revised the manuscript to strictly distinguish between the two concepts.

7. Since the electronic structure of a molecule is determined by its wavefunction, the term local electronic distribution may be more appropriate than electronic inhomogeneity.

We have now modified the term electronic inhomogeneity to local electronic distribution. Please see **REV#10** in the abstract.

8. The manuscript states that "A photocurrent of ~100 fA at an 80-MHz repetition rate corresponds to ~0.008 electrons per laser pulse". However, because STM detects only the DC component of the current, the AC component induced by photoexcitation may not be directly measurable. In that regard, clarification on whether this estimate represents a physically meaningful quantity would be appreciated.

We thank the reviewer for this comment. We agree that the STM detects the time-averaged DC current rather than instantaneous AC signal. We cited the value of "~0.008 electrons per pulse" not to imply AC detection, but to highlight the low probability of a photo-induced tunneling event occurring for any given laser shot.

We have now modified the relevant sentence in the main-text as "*Because the ~ 80 MHz repetition rate of the laser pulses far exceeds the ~1 kHz bandwidth of the preamplifier used in the STM, the discrete photon-induced tunneling events are integrated into the measured DC tunneling current. A measurable photocurrent of 100 fA implies a statistical average of ~0.008 tunneling events per laser pulse*". Please see **REV#11** in the main-text on page 3.

Response to the comments of Reviewer #1

Reviewer #1 (Remarks to the Author):

The authors have revised the paper and reacted to the comments of the reviewers. I still believe that the paper is of high experimental quality and deserves publication.

We would like to sincerely thank the reviewer once again for their kind words and for the thorough evaluation of our manuscript. We have made all possible efforts to improve the clarity of all the points raised and to meet the expectations of the reviewer. Below, we provide a point-by-point response to the comments raised.

In response to my comments the authors removed a part of the supplementary material concerning the microscopic details of the plasmon-exciton interaction and introduced a new simplified way to treat the local interactions. Unfortunately, I think that my comments were not understood by the authors and their modifications concerning the use of the differential dipole density ("transition dipole density") \mu and its use to describe the localized plasmon-exciton interaction in Supp. Eq. 18 made the theory even less physically meaningful. In a separate document, I therefore attach a brief description of the technical problems that I see with the approach of the authors as well as a suggestion how to correct it. This issue concerns both the main text (Rev#2) and, primarily, the Supp. Mat.

The authors are right that "transition dipole density" can be defined and is accessible in quantum chmistry codes. However, this does not have any implications regarding its physical meaning, particularly in the context of STM. The excistence of literature actually using the "transition dipole density" by no means shows that its use is appropriate, certainly not in the present context.

To be clear, I don't think that the use of the "transition dipole density" is a matter of taste, I am convinced that, when dealing with inhomogeneous plasmonic field, it is strictly incorrect (in the mathematical sense). I therefore cannot recmmend the paper for publication unless this issue is satisfactorily resolved (for the sake of the quality of the text and preventing the spread of misconceptions). If the authors show me a rigorous mathematical proof (i.e. not just a reference to external resources) that the approach using "transition dipole density" is correct in the present context of inhomogeneous plasmonic field, I will be happy to accept its use. Otherwise I must insist on using a different, physically meaningful, approach.

The reviewer is strictly right. With the aim of going beyond the simplest description based on just the densities of the initial and final states, and incorporating the effect of an external field in the simplest possible way, we calculated "transition dipole densities", which are standard in quantum chemistry for gas-phase calculations but that, as the referee points out, may still be far away from what is really needed to obtain the true "effective oscillator strength". Nevertheless, our results were sound because the conclusions were rather independent on the level of description of this effective oscillator strength. In any case, we agree with the reviewer that every possible source of error or confusion should be avoided, so, in the new version of the manuscript, we decided to remove the discussion on μ and its usage altogether, and based everything on the transition density, as

suggested by the reviewer, which is not affected by the ambiguity of the system or reference choice. Since we needed a way of computing the optical activity of the molecules in a locally varying electric field, following the suggestion of the reviewer, we computed the electric potential due to the presence of two point dipoles. Please, have a look at the new paragraph included in the Supplementary Information (page #17). We found a qualitative agreement with the experimental results. The results of these calculations corroborate that the presence of the STM tip and its precise placing over the molecule enhances (or quenches) certain allowed as well as forbidden optical transitions, as observed experimentally. All these changes are implemented in the new version of the manuscript, please see new Fig. 4 in the main-text (and the corresponding text in page #7) and sections on 'transition density' and 'two point-dipole modelling' in the Supplementary Information.

I appreciate the additions to the discussion of the open-shell character of the molecule and the triplet character of the dimer. I also appreciate the addition of transition densities in the Supp. Mat.

Thank you.

Regarding my question on how abrupt/smooth the changes in photocurrent with external bias are: I am not sure if the authors fully appreciate the references that are cited in the answer (Nature 603, 829–834 (2022); Nature Communications 16 (1), 6039 (2025)). If the photocurrents simply came from the relative alignmnent of the electronic levels of the molecule with the Fermi level of the electrodes, one would expect sharp variations of the photocurrents upon reaching certain theresholds. As far as I understand, it is the substrate reorganization that smears out these thresholds and thus enables the fine-tuning of the balance between various photocurrent channels (related to the phonon-blockade effect). Maybe the authors could comment on this effect in the main text as well, as this is a subtle, but important, detail that could improve the awareness of the reader and prevent the reader from falling for misconceptions.

We thank the reviewer for the comment. The issue of reorganization of the substrate energies following photon-induced tunneling has been discussed extensively in the cited papers. We have not delved into it to avoid unnecessary repetition.

The addition of rev#3 is appreciated.

Thank you.

There are typos in Eq. 3 of the main text and Supplementary Eq. 10, 11. I also ask the authors to check for the presence of factors of "N" in these equations. I suspect that it might be missing (depending on how the quantities are normalized).

We have carefully checked these equations and corrected the typos. Furthermore, the normalization of N was indeed missing. In one of the transformations of the theory, the "sum over the electrons" was dropped but we did not add the N normalization that replaces it. Thank you again for pointing this out.

Page 6 of the main text: SOMO and SUMO orbitals -> SUMO and SUMO (O stands for orbital).

Thank you for pointing this out. We have corrected this.

In the original version of the supplementary material the authors suggested that an effective oscillator strength can be defined as [supp. Eq. (18)]:

$$\tilde{f}^j \propto \left|\iiint_{-\infty}^{\infty} \boldsymbol{E}(x,y,z) \cdot \boldsymbol{\mu}^j(x,y,z) \mathrm{d}x\, \mathrm{d}y\, \mathrm{d}z\right|^2, \tag{1}$$

where $\boldsymbol{\mu}^j(x,y,z)$ is the differential form of the dipole matrix element depending on the cartesian coordinates x, y, z, and $\boldsymbol{E}(x,y,z)$ is the spatially inhomogeneous electric field of the plasmon. The dipolar matrix element is defined as

$$\boldsymbol{d}^{ij} = -e\sum_q \int \Psi_i^*(\boldsymbol{r}_1, \dots \boldsymbol{r}_n)\boldsymbol{r}_q\Psi_j(\boldsymbol{r}_1, \dots \boldsymbol{r}_n)\, \mathrm{d}^3\boldsymbol{r}_1 \dots \mathrm{d}^3\boldsymbol{r}_N = -e\int \rho_{ij}(\boldsymbol{r})\boldsymbol{r}\, d^3\boldsymbol{r}, \tag{2}$$

with $-e$ being the electron charge, $\Psi_j(\boldsymbol{r}_1, \dots \boldsymbol{r}_n)$ being the many-particle wave function of the electronic system, $\boldsymbol{r}_q$ being the position vector of electron q, and $\rho_{ij}(\boldsymbol{r})$ being the transition density depending on the single spatial coordinate $\boldsymbol{r}$.

The differential form of it is then (follows from the definitions in the manuscript):

$$\boldsymbol{\mu}^j(\boldsymbol{r}) = \rho_{ij}(\boldsymbol{r})\boldsymbol{r}. \tag{3}$$

This expression depends on the choice of the origin of coordinates and is clearly ambiguously defined as a local quantity. Only the integral of it over whole space is well defined.

In the limit of homogeneous electric field, one often expresses the light-matter coupling g, related to $\tilde{f}^j$ by $\tilde{f}^j \propto |g|^2$, as:

$$g \approx -\boldsymbol{E} \cdot \boldsymbol{d}^{ij}, \tag{4}$$

which can be derived from the expression valid on the scale of the atomistic STM junction:

$$g = -e\int \rho_{ij}(\boldsymbol{r})\phi_{pl}(\boldsymbol{r})\, d^3\boldsymbol{r}, \tag{5}$$

where $\phi_{pl}(\boldsymbol{r})$ is the quasi-electrostatic potential representing approximately the distribution of the quasi-static electric field in the plasmonic cavity. Expression (5) originates directly from the effect of the Coulomb interaction and can be derived from the many-body theory of plasmon-exciton interaction.

Does Eq. (5) agree with Eq. (1) presented by the authors? Eq. (1) would imply that

$$g = e\int \rho_{ij}(\boldsymbol{r})\boldsymbol{r} \cdot \nabla\phi_{pl}(\boldsymbol{r})\, d^3\boldsymbol{r}, \tag{6}$$

which can only be identical to Eq. (5) if $\boldsymbol{r} \cdot \nabla\phi_{pl}(\boldsymbol{r}) = -\boldsymbol{r} \cdot \boldsymbol{E}(\boldsymbol{r}) = -\phi_{pl}(\boldsymbol{r})$ or if $\nabla\big(\rho_{ij}(\boldsymbol{r})\boldsymbol{r}\big) = \rho_{ij}(\boldsymbol{r})$, where the latter can be obtained by integrating Eq.(6) by parts. **Clearly, none of these equalities hold for spatially inhomogeneous fields so Eq. (5) is not compatible with the suggestion shown in Eq. (1).**

Since Eq. (5) directly proceeds from the Coulomb integral (and has been verified in the past), one must question the validity of Eq. (1) in situations where the plasmonic field is strongly spatially inhomogeneous.

In the new version of the supplementary material the authors propose a new expression for the oscillator strength:

$$\tilde{f}^j \propto \left|\int_\Omega \boldsymbol{\mu}^j(x,y,z)\, \mathrm{d}x\mathrm{d}y\mathrm{d}z\right|^2, \tag{7}$$

where the integration is performed over a small volume Ω reflecting the position of the tip. This expression would only be applicable if the electric field were nonzero within the integration volume, which is nonphysical as the field varies smoothly in space.

Suggestion:

The authors could directly use Eq. (5) to calculate the tip-molecule coupling – to that end they only need to extract the transition density of the molecule and calculate a suitable quasi-electrostatic potential of the plasmonic cavity (similarly to how they expressed the electric field in the original version of the supplementary material). This would be a physically meaningful definition of the generalized oscillator strength.
